# CPT1a-Dependent Long-Chain Fatty Acid Oxidation Contributes to Maintaining Glucagon Secretion from Pancreatic Islets

**DOI:** 10.1016/j.celrep.2018.05.035

**Published:** 2018-06-13

**Authors:** Linford J.B. Briant, Michael S. Dodd, Margarita V. Chibalina, Nils J.G. Rorsman, Paul R.V. Johnson, Peter Carmeliet, Patrik Rorsman, Jakob G. Knudsen

**Affiliations:** 1 Oxford Centre for Diabetes, Endocrinology and Metabolism, Radcliffe Department of Medicine, University of Oxford, Oxford OX3 7LE, UK; 2 Department of Computer Science, University of Oxford, Oxford OX1 3QD, UK; 3 Department of Physiology, Anatomy & Genetics, University of Oxford, Parks Road, Oxford OX1 3PT, UK; 4 Faculty of Health and Life Sciences, Coventry University, Coventry CV1 5FB, UK; 5 Oxford National Institute for Health Research, Biomedical Research Centre, Churchill Hospital, Oxford OX3 7LJ, UK; 6 Laboratory of Angiogenesis and Vascular Metabolism, VIB-KU Leuven Center for Cancer Biology, Leuven, Belgium; 7 Metabolic Research, Department of Neuroscience and Physiology, Sahlgrenska Academy, University of Göteborg, Box 433, 405 30 Göteborg, Sweden

**Keywords:** islet, metabolism, glucose, Ca2+, KATP, liver, fasting

## Abstract

Glucagon, the principal hyperglycemic hormone, is secreted from pancreatic islet α cells as part of the counter-regulatory response to hypoglycemia. Hence, secretory output from α cells is under high demand in conditions of low glucose supply. Many tissues oxidize fat as an alternate energy substrate. Here, we show that glucagon secretion in low glucose conditions is maintained by fatty acid metabolism in both mouse and human islets, and that inhibiting this metabolic pathway profoundly decreases glucagon output by depolarizing α cell membrane potential and decreasing action potential amplitude. We demonstrate, by using experimental and computational approaches, that this is not mediated by the K_ATP_ channel, but instead due to reduced operation of the Na^+^-K^+^ pump. These data suggest that counter-regulatory secretion of glucagon is driven by fatty acid metabolism, and that the Na^+^-K^+^ pump is an important ATP-dependent regulator of α cell function.

## Highlights

• Glucagon secretion in low glucose is maintained by CPT1a-dependent FAO
• Loss of CPT1a-dependent FAO in mouse and human islets decreases glucagon secretion
• CPT1a-dependent FAO maintains glucagon secretion by supplying ATP to the Na^+^-K^+^-ATPase
• CPT1a-dependent FAO contributes to the counter-regulatory secretion of glucagon

## Graphical Abstract

**Figure:**
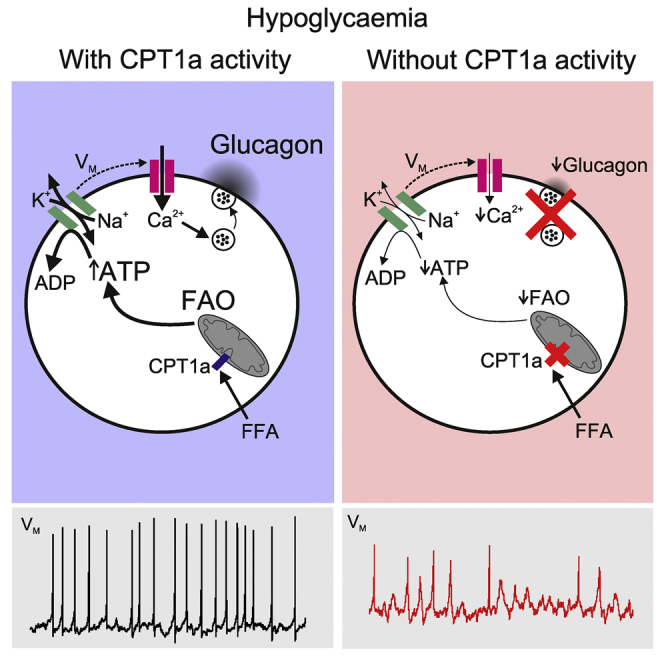

## Teaser

Glucagon is secreted from pancreatic α cells in hypoglycemic conditions. Briant et al. demonstrate that this response is fueled by fatty acid oxidation. The energy generated by oxidation is used to maintain membrane potential dynamics, action potential morphology, and Na^+^-K^+^ pump activity.

## Introduction

Type 2 diabetes mellitus (T2DM) is a metabolic disorder characterized by hyperglycemia, insulin resistance, and insufficient insulin secretion from islet β cells (Leahy, 2005). However, it is becoming increasingly apparent that over-secretion of glucagon from pancreatic α cells also contributes to the increased hepatic glucose production and associated hyperglycemia in T2DM. The abnormalities in glucagon secretion in T2DM include both loss of adequate suppression under hyperglycemic conditions (Cryer, 2002, Cryer, 2008, Cryer et al., 2003, Dunning et al., 2005, D’Alessio, 2011, Unger and Cherrington, 2012) and insufficient release during episodes of hypoglycemia (Gerich et al., 1973, Bolli and Perriello, 1990, Shamoon et al., 1994).

Despite the centrality of glucagon in the etiology of T2DM, the mechanisms by which glucagon secretion is regulated at low glucose have not been fully elucidated. Several paracrine processes have been demonstrated to alter glucagon secretion (Gromada et al., 2007, Gylfe, 2013, Gylfe, 2016, Caicedo, 2013, Gylfe and Gilon, 2014). However, in low glucose, glucagon secretion is controlled by mechanisms intrinsic to the α cell (Vieira et al., 2007, Zhang et al., 2013).

The β cell is tailored to deal with conditions of high glucose: the high-K_m_ GLUT transporter (GLUT2), high-K_m_ glucokinase, and high respiratory capacity in β cells result in a robust insulin secretory response to hyperglycemia (Heimberg et al., 1995, Heimberg et al., 1996, Díaz et al., 2007). In the α cell, glucokinase activity (Heimberg et al., 1996) and glycolytic flux (Heimberg et al., 1995) are comparable with that of β cells. However, glucose oxidation (Schuit et al., 1997, Detimary et al., 1998), as well as glucose-induced ATP (Detimary et al., 1998, Ishihara et al., 2003, Ravier and Rutter, 2005, Li et al., 2015), FADH_2_ (Quesada et al., 2006), and NAD(P)H (Quoix et al., 2009) production are all lower in α cells. Despite α cells having a lower capacity to produce ATP in response to glucose, α cells are still able to retain secretory function at low or even in the complete absence of glucose (De Marinis et al., 2010, Olsen et al., 2005, Salehi et al., 2007, Vieira et al., 2007).

Some tissues have an obligatory need for glucose as an energy substrate (Bélanger et al., 2011). However, many tissues in the body act as “omnivores” using a variety of carbon substrates as energy sources to sustain sufficient ATP generation. β-oxidation of free fatty acids (FFAs) is a major energy source in the heart (Most et al., 1969, Lopaschuk et al., 2010), muscle (Rasmussen et al., 2002), and liver (Ontko, 1972). In the islet, FFAs are known to regulate glucose-induced insulin secretion independently of β-oxidation via G-protein-coupled receptor GPR40 signaling (Itoh et al., 2003). In the α cell, less is known about the role of FFAs in regulating glucagon secretion. Although short-term exposure to supra-physiological levels of FFA have been demonstrated to increase glucagon secretion (Olofsson et al., 2004), this may be due to GPR40 signaling (Kristinsson et al., 2017), rather than β-oxidation. Circulating FFAs are essential for maintaining systemic energy homeostasis during hypoglycemia (Hue and Taegtmeyer, 2009). Given that β-oxidation of FFAs can provide substantial amounts of ATP, it may be that α cells utilize this energy source, oxidizing FFAs to maintain secretory function in conditions of hypoglycemia.

Here, we show that the oxidation of long-chain FFAs in α cells contributes to maintaining glucagon secretion under hypoglycemic conditions. We also provide a mechanism by which fatty acid oxidation (FAO) energizes glucagon secretion in counter-regulatory conditions.

## Results

### Glucagon Secretion from Mouse α Cells Depends on FAO

**Figure 1:**
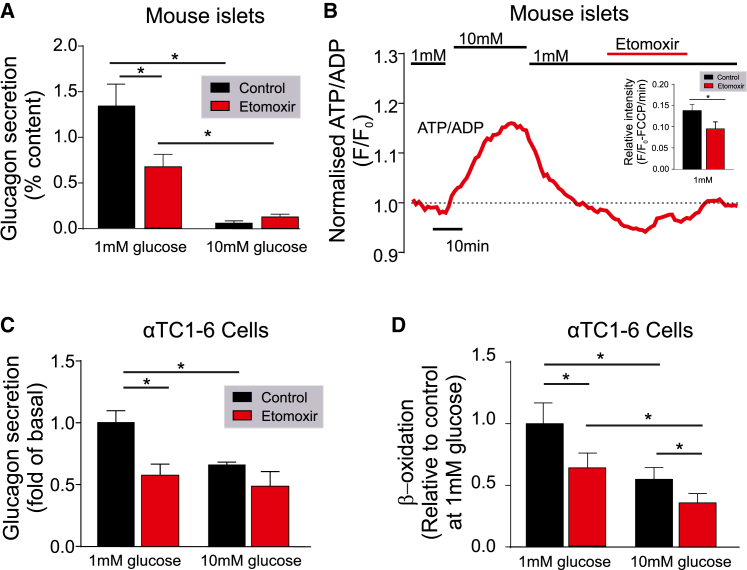
Blocking FFA Transport Pharmacologically or by CPT1a Knockdown Reduces Glucagon Secretion in Mouse α Cells and αTC1-6 Cells (A) Glucagon secretion from WT mouse islets at 1 and 10 mM glucose with or without etomoxir (100 μM) reduced glucagon secretion (n = 3 mice). (B) ATP production in WT mouse islets during exposure to 1 or 10 mM glucose, as well as 1 mM glucose and etomoxir (100 μM) (n = 4 mice). (C) Glucagon secretion from αTC1-6 cells at 1 and 10 mM glucose with or without etomoxir (100 μM) (n = 3). (D) β-Oxidation measured using [^3^H]palmitate in αTC1-6 cells at 1 and 10 mM glucose with or without etomoxir (100 μM) (n = 6 observations, from 2 experiments). All data are represented as mean ± SEM. Paired t test with Tukey post hoc or two-way ANOVA with Student-Newman-Keuls post hoc; ^∗^p < 0.05. See also Figure S1.

We first explored the dependence of glucagon secretion under hypoglycemic conditions on FAO (Figure 1). Carnitine palmitoyltransferase 1 (CPT1) is a mitochondrial transmembrane enzyme responsible for the formation of acyl-carnitine from long-chain acyl-coenzyme A's (CoA), which can subsequently be transported into the mitochondria and used for β-oxidation. This enzyme is considered rate limiting for β-oxidation of long-chain fatty acids (LCFAs; Kim et al., 2000, Stephens et al., 2007). When mouse islets were exposed to low (1 mM) glucose, pharmacological blockade of CPT1 with etomoxir (100 μM) reduced glucagon secretion by ∼40% (p < 0.001; Figure 1A) and decreased the cytoplasmic ATP/ADP ratio (p < 0.0001; Figure 1B). This was via a direct effect on α cells, because insulin signaling was not altered (p = 0.84; Figure S1). Etomoxir also reduced glucagon secretion by ∼40% in αTC1-6 cells at 1 mM glucose (p = 0.005; Figure 1C). This was due to a ∼40% reduction in β-oxidation (p = 0.017; Figure 1D). These data show that FAO contributes to glucagon secretion in hypoglycemic conditions.

### CPT1a is Essential for Fat Oxidation and Glucagon Secretion in α Cells

**Figure 2:**
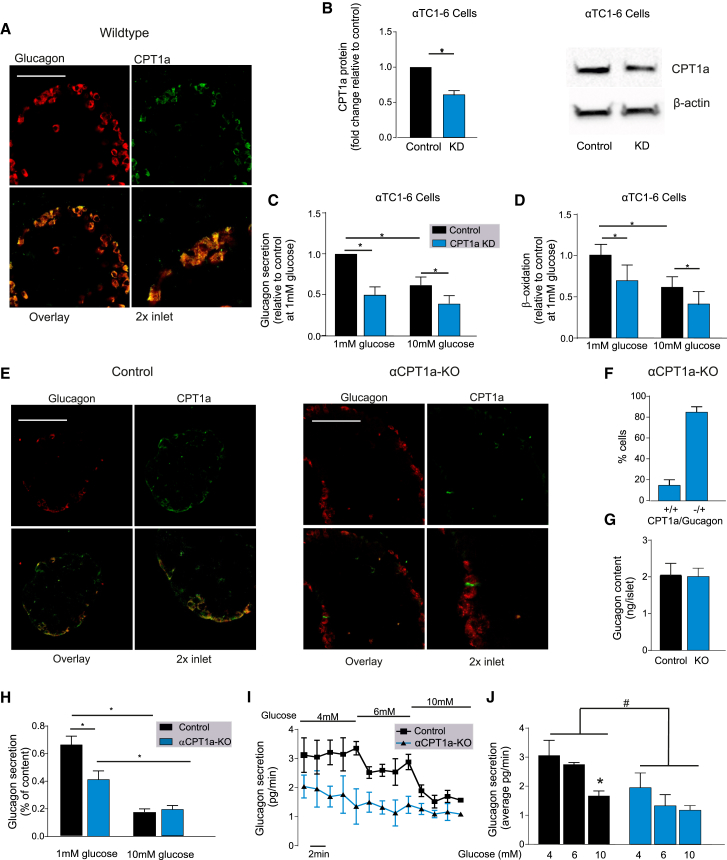
Knockout of CPT1a Specifically in α Cells Reduces Glucagon Secretion (A) Immunofluorescence for glucagon (red) and CPT1a (green) in WT mouse islets from pancreatic slides. Scale bar, 50 μM. (B) Knockdown (KD) of CPT1a in αTC1-6 cells treated with either scrambled small interfering RNA (siRNA; control) or siRNA targeting *Cpt1a* mRNA (note the ∼40% reduction) (n = 6 observations from 3 experiments). (C) Glucagon secretion in αTC1-6 cells at 1 and 10 mM glucose treated with either scrambled siRNA (control) or siRNA targeting *Cpt1a* mRNA (n = 3). (D) β-Oxidation measured using [^3^H]palmitate in αTC1-6 cells at 1 and 10 mM glucose treated with either scrambled siRNA (control) or siRNA targeting *Cpt1a* mRNA (n = 6 observations, from 2 experiments). (E) Immunofluorescent detection of glucagon (red) and CPT1a (green) in isolated islets from α cell-specific knockout of *Cpt1*a (αCPT1a-KO) and littermate control mice. Scale bar, 50 μM (n = 5). (F) Percentage (%) of cells that show co-localization of CPT1a and glucagon in αCPT1a-KO mice (n = 5 islets, 3 mice, 145 cells). (G) Glucagon content in isolated islets from control and αCPT1a-KO (KO) islets (n = 6). (H) Glucagon secretion from isolated islets from control and αCPT1a-KO mice at 1 and 10 mM glucose (n = 6). (I) Glucagon secretion during perfusion in control (n = 3) and αCPT1a-KO (n = 3) mice at 4, 6, and 10 mM glucose. (J) Average glucagon secretion from control (n = 3) and αCPT1a-KO mice (n = 3) at 4, 6, and 10 mM glucose, calculated from the last 8–10 min in each condition. Two-way ANOVA with post hoc: ^#^p < 0.05 versus control; ^∗^p < 0.05 versus 4 mM glucose. Paired t test with Tukey post hoc or two-way ANOVA; ^∗^p < 0.05. See also Figure S1.

We investigated the expression of CPT1 in α cells and found a high degree of co-localization between glucagon and the liver CPT1 isoform (CPT1a) in wild-type (WT) mouse islets (Figure 2A). The muscle CPT1 isoform (CPT1b) is also known to be expressed in α cells, but to a lower degree than CPT1a (Benner et al., 2014). Knockdown of *Cpt1a* in αTC1-6 cells (40% reduced protein content; Figure 2B) resulted in a decrease in glucagon secretion (p = 0.003; Figure 2C) and β-oxidation (p = 0.033; Figure 2D) at 1 mM glucose, consistent with our pharmacological blockade of CPT1 with etomoxir (Figure 1).

The αTC1-6 cell line is an artificial system, and so to understand the implications of reduced CPT1a expression in whole islets, we generated an α cell-specific *Cpt1a* knockout (αCPT1a-KO) mouse. Islets from these mice have no CPT1a expression in 85% ± 2% of the α cells (Figures 2E and 2F). Knockout of *Cpt1a* reduced glucagon secretion by ∼40% at 1 mM glucose (p < 0.001; Figure 2H), without affecting glucagon content (p = 0.937; Figure 2G) or glucagon secretion at 10 mM glucose (p = 0.64; Figure 2H). Insulin secretion was not affected by the loss of *Cpt1a* in α cells (p = 0.43; Figure S1B). Blood glucose is unlikely to reach 1 mM *in vivo*; therefore, we investigated glucagon secretion *in situ* from control and αCPT1a-KO by perfusion of the whole pancreas with 4, 6, and 10 mM glucose. Glucagon secretion from αCPT1a-KO was significantly lower compared with control mice (p = 0.003; Figures 2I and 2J), and only control mice responded by significantly lowering glucagon secretion in response to 10 mM glucose (p = 0.045; Figures 2I and 2J). These data suggest that *Cpt1a* knockout reduces glucagon secretion from mouse islets in conditions of hypoglycemia.

### *Cpt1a* Knockout Reduces Fasting Plasma Glucose

**Figure 3:**
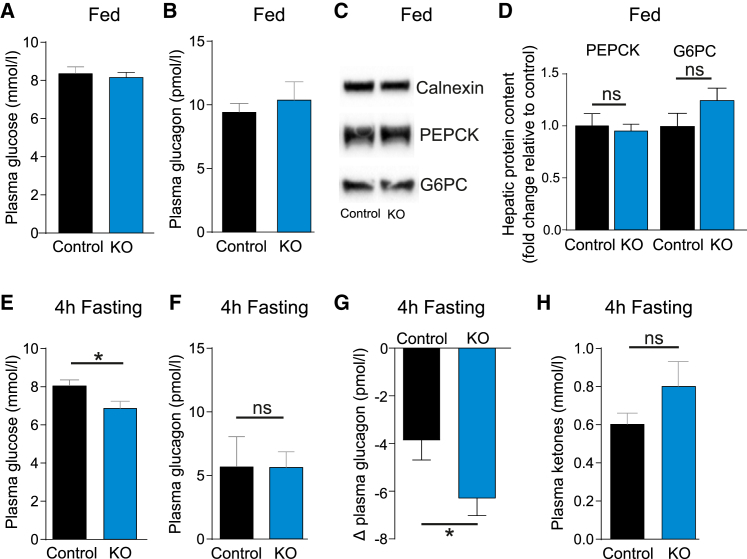
*Cpt1a* Knockout Reduces Fasting Plasma Glucose (A) Plasma glucose in fed control and αCPT1a-KO (KO) mice (n = 14 ). (B) Plasma glucagon in fed control and KO mice (n = 4–5). (C) Representative blots of PEPCK, G6PC, and calnexin in fed control and KO mice. (D) Protein content of hepatic PEPCK and G6PC in fed control and KO mice (n = 3–5). (E) Plasma glucose in control and KO mice following a 4-hr fast (n = 10–12). (F) Plasma glucagon in control and KO mice following a 4-hr fast (n = 4–5). (G) Difference between 4 hr fasted and fed plasma glucagon in control and KO mice (n = 4–5). (H) Plasma ketone bodies in control and KO mice following a 4-hr fast (n = 7–9). All data are represented as mean ± SEM. Paired t test with Tukey post hoc; ^∗^p < 0.05. ns, not significant. See also Figure S1.

To test whether FAO contributes to the counter-regulatory response to hypoglycemia *in vivo*, we measured plasma glucose and glucagon in αCPT1a-KO mice (Figure 3). Knockout of *Cpt1a* did not change fed plasma glucose (p = 0.669; Figure 3A), glucagon (p = 0.608; Figure 3B), or hepatic protein content of the gluconeogenic enzymes phosphoenolpyruvate carboxykinase (PEPCK; p = 0.999) and glucose 6-phosphatase (G6PC; p = 0.25; Figures 3C and 3D). Following a 4-hr fast, plasma glucose was decreased in αCPT1a-KO mice compared with control mice (p = 0.03; Figure 3E). Despite plasma glucose being lower in αCPT1a-KO compared with control mice following a 4-hr fast, plasma glucagon was no different between the two genotypes (p = 0.987; Figure 3F). In line with previous studies (Allister et al., 2013), 4 hr of fasting seemed to reduced plasma glucagon in the fed state in both αCPT1a-KO and control mice. The change in plasma glucagon relative to the fed state was greater in αCPT1a-KO compared with control mice (p = 0.029; Figure 3G), indicating a change in the regulation of circulating glucagon levels. We next tested whether ketone body metabolism could contribute to the maintenance of glucagon secretion in WT islets during hypoglycemia. Treating WT islets with 3-hydroxybuterate (0.5 mM) reduced glucagon secretion at 1 mM glucose (p = 0.0002; Figure S1J). This is in agreement with studies from perfused rat pancreas and human subjects (Ikeda et al., 1987, Quabbe et al., 1983) where glucagon secretion was decreased or unaffected by ketones bodies. It is therefore unlikely that ketone metabolism in α cells of the αCPT1a-KO mice could have compensated for the reduced FAO. In support of this, the concentration of fasted plasma ketone bodies was not different in αCPT1a-KO and control mice (p = 0.21; Figure 3H). These data demonstrate that FAO in α cells may contribute to maintaining blood glucose during a 4-hr fast, and that this response is impaired in αCPT1a-KO mice.

### Disruption of FAO Changes Electrical Activity in α Cells

α cells are electrically active, utilizing these electrical signals to drive glucagon secretion (Barg et al., 2000, Gromada et al., 2007, MacDonald et al., 2007, Jacobson et al., 2009, Ramracheya et al., 2010, Zhang et al., 2013, Gylfe and Gilon, 2014). Given that inhibition of FAO decreased glucagon secretion (Figure 2), we hypothesized that this was due to an effect on α cell membrane potential.

**Figure 4:**
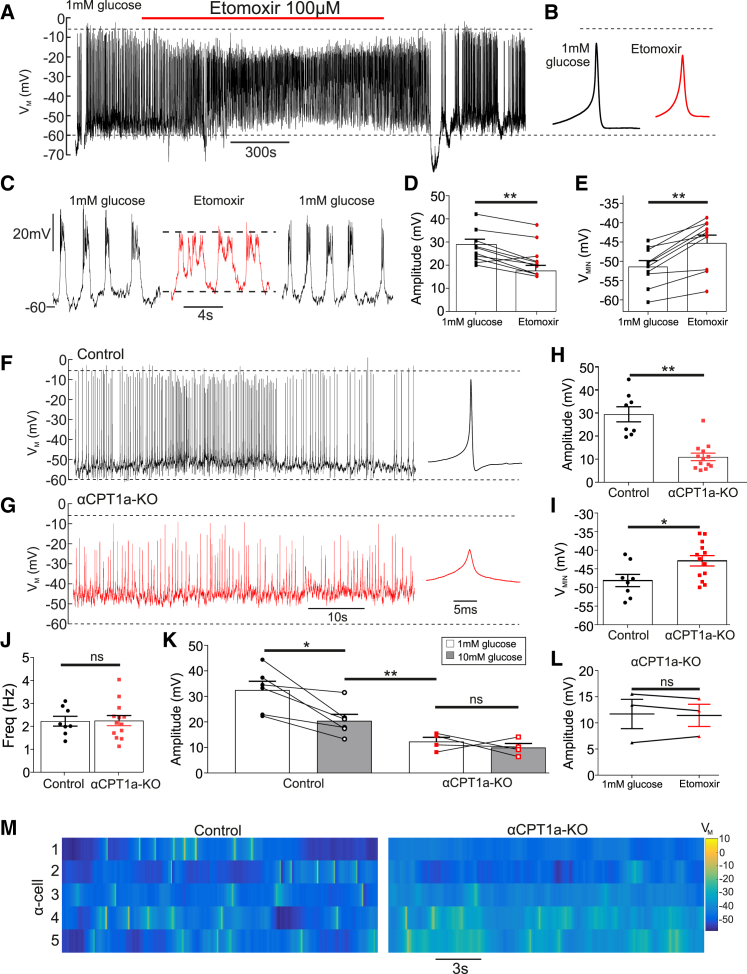
Disruption of FFA Transport by CPT1a Reduces Action Potential Amplitude in Mouse α Cells (A) Electrical activity in WT α cell at 1 mM glucose with or without etomoxir (100 μM) (10 islets from 6 mice). (B) Average action potential waveform for (A), in etomoxir compared with 1 mM glucose, measured over the entire experimental condition. (C) Expanded view on 1 mM glucose and etomoxir conditions for (A). (D) Action potential amplitude in WT α cells at 1 mM glucose with or without etomoxir (100 μM) (10 islets from 6 WT mice). (E) Minimum membrane potential (V_MIN_) in WT α cells at 1 mM glucose with or without etomoxir (100 μM) (10 islets from 6 mice). (F) Electrical activity in control α cell at 1 mM glucose. (G) Electrical activity in αCPT1a-KO α cell at 1 mM glucose. (H) Action potential amplitude in α cells from αCPT1a-KO compared with control at 1 mM glucose (8 control islets for 4 mice and 13 αCPT1a-KO islets for 4 mice). (I) Minimal potential (V_MIN_) in α cells from αCPT1a-KO mice compared with control at 1 mM glucose (8 control islets for 4 mice and 13 αCPT1a-KO islets for 4 mice). (J) Firing frequency in control and αCPT1a-KO islets at 1 mM glucose (8 control islets for 4 mice) and 13 αCPT1a-KO islets for 4 mice). (K) Action potential amplitude in α cells from control and αCPT1a-KO islets at 1 and 10 mM glucose (6 control islets for 4 mice and 6 αCPT1a-KO islets for 4 mice). (L) Action potential amplitude in α cells from αCPT1a-KO islets at 1 mM glucose with or without etomoxir (100 μM) (3 islets from 3 mice for each genotype). (M) Raster plots demonstrating robust action potentials in 5 control α cells, and weaker action potentials in 5 α cells from αCPT1a-KO islets. Paired t test with Tukey post hoc; ^∗^p < 0.05; ^∗∗^p < 0.01; ^∗∗∗^p < 0.001. See also Figures S1 and S2.

Inhibition of FAO resulted in a reduction in action potential (AP) amplitude in mouse α cells (Figure 4). Etomoxir (100 μM) reduced AP amplitude (p < 0.0001; Figures 4A–4D) and depolarized the minimum membrane potential by 7 mV (p < 0.001; Figure 4E). Etomoxir also reduced the amplitude of intracellular Ca^2+^ oscillations (Figure S2). The effect of pharmacological blockade of FAO on membrane potential in α cells was mirrored in αCPT1a-KO mice (Figures 4F–4M). α cells from αCPT1a-KO mice had a lower AP amplitude compared with control mice (p < 0.0001; Figure 4H). Minimal membrane potential in αCPT1a-KO mice was depolarized compared with controls (p = 0.026; Figure 4I). The frequency of AP firing was not changed (p = 0.94; Figure 4J), suggesting that disruption of FAO reduces glucagon secretion by reducing AP amplitude, rather than firing frequency. High glucose (>6 mM) is known to decrease glucagon secretion by reducing AP amplitude in α cells by ∼17 mV (Zhang et al., 2013). Consistent with this, in control mice, high glucose (10 mM) decreased AP amplitude by 12 ± 2 mV compared with 1 mM glucose (p = 0.014; Figure 4K). In contrast, 10 mM glucose did not decrease AP amplitude in αCPT1a-KO mice (p = 0.94). Furthermore, the reduction in AP amplitude in αCPT1a-KO at 1 mM glucose was significantly greater than in control islets due to 10 mM glucose (p = 0.005). Therefore, the suppression of AP amplitude by FAO disruption is physiologically significant.

### Disruption of FAO Alters α Cell Membrane Potential via a K_ATP_-Independent Mechanism

We investigated whether the effect of CPT1 inhibition was due to a decrease in K_ATP_ channel conductance (G_KATP_). First, we noted that our pharmacological data (Figure 4) appeared incompatible with a K_ATP_-dependent mechanism because etomoxir decreased intracellular ATP (*[ATP]*_*i*_) (Figure 1B), which would result in an increase in G_KATP_, consequently hyperpolarizing the membrane. Nevertheless, because the K_ATP_ channel is crucial to glucagon secretion (MacDonald et al., 2007, Zhang et al., 2013), we wanted to investigate the dependence (if any) of the effect on K_ATP_ channel activity.

**Figure 5:**
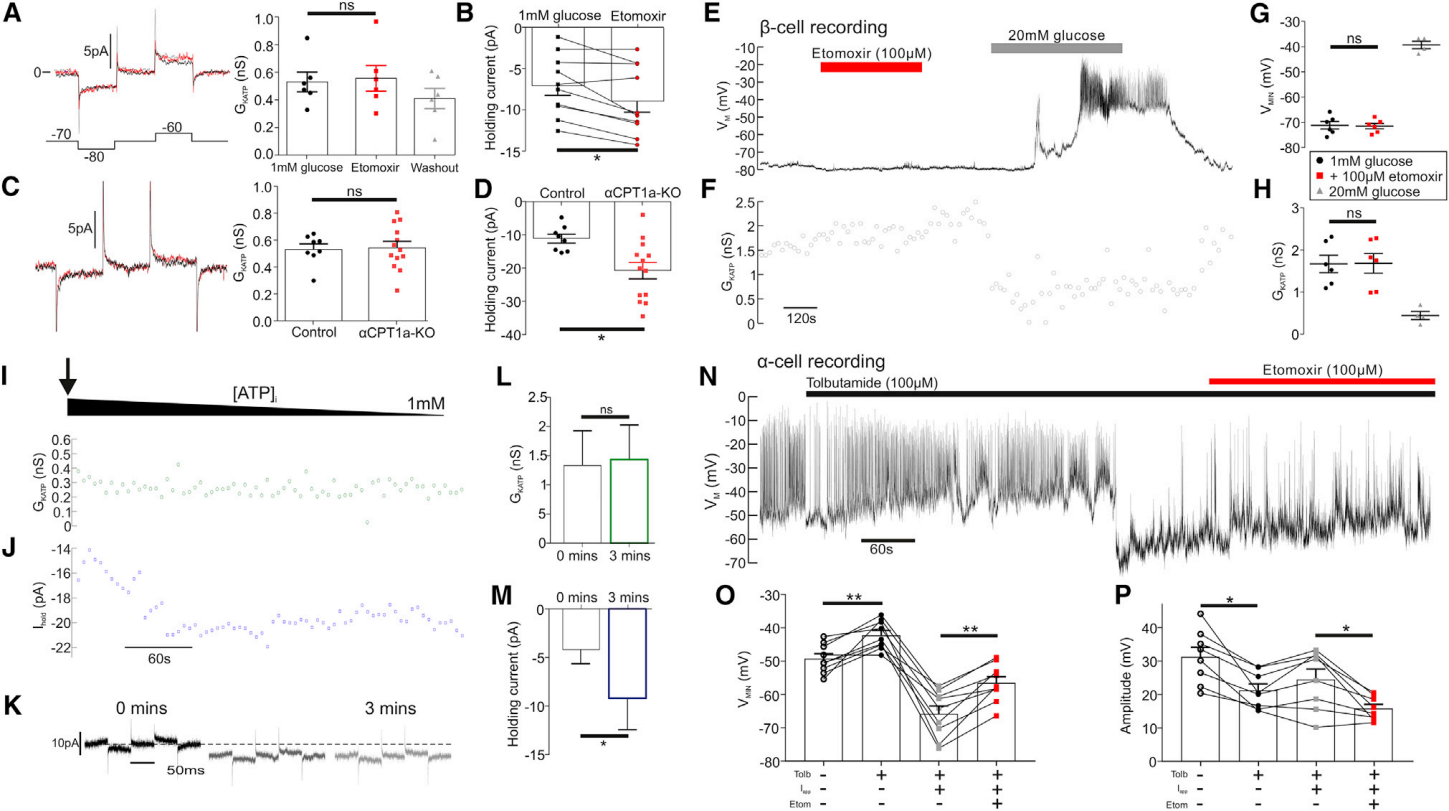
Inhibition of CPT1a Disrupts α Cell Membrane Potential via a K_ATP_-Independent Mechanism (A) G_KATP_ in wild-type (WT) mouse α cells at 1 mM glucose with or without etomoxir (100 μM) (6 islets, 5 mice). (B) Holding current in WT mouse α cells at 1 mM glucose with or without etomoxir (100 μM) (11 islets, 6 mice). (C) G_KATP_ in α cells from αCPT1a-KO mice compared with control at 1 mM glucose (8 control islets from 4 mice and 13 αCPT1a-KO islets from 4 mice). (D) Holding current in α cells from αCPT1a-KO mice compared with control at 1 mM glucose (8 control islets for 4 mice and 13 αCPT1a-KO islets for 4 mice). (E) Electrical activity in WT β cell at 1 mM glucose with or without etomoxir (100 μM) and at 20 mM glucose. (F) G_KATP_ in WT β cell at 1 mM glucose with or without etomoxir (100 μM) and at 20 mM glucose. (G) Grouped data of V_M_ recording in β cells following etomoxir and 20 mM glucose application (6 islets from 6 WT mice). (H) Grouped data of G_KATP_ recording in β cells following etomoxir and 20 mM glucose application (6 islets from 6 WT mice). (I) To mimic the effects of etomoxir in 1 mM glucose on *[ATP]*_*i*_, we artificially reduced *[ATP]*_*i*_ by setting the pipette concentration (1 mM) to be lower than the putative *[ATP]*_*i*_ in 1 mM glucose (>1 mM; Detimary et al., 1998) and measured G_KATP_. The arrow denotes when whole cell was achieved, which initiates the run-down of *[ATP]*_*i*_ from the endogenous concentration to the pipette concentration. (J) Holding current (I_hold_) from (I). (K) G_KATP_ and I_hold_ after 0, 1, and 3 min of whole-cell recording (I). (L) Grouped data, recording G_KATP_ during artificial run-down of *[ATP]*_*i*_ (4 WT islets, 4 mice). (M) Grouped data, recording I_hold_ during artificial run-down of *[ATP]*_*i*_ (4 WT islets, 4 mice). (N) Membrane potential recording from a WT α cell. Tolbutamide was applied to maximally open K_ATP_ channels. A negative current was then injected to hyperpolarize the α cell. Etomoxir was still able to depolarize α cells. (O) Grouped data for change in minimum potential (V_MIN_) (n = 8 islets, n = 4 mice). (P) Grouped data for change in action potential amplitude (n = 8 islets, n = 4 mice). Paired t test with Tukey post hoc or two-way ANOVA; ^∗^p < 0.05; ^∗∗^p < 0.01; ^∗∗∗^p < 0.001. See also Figures S1 and S2.

We measured G_KATP_ in response to inhibition of CPT1a (Figure 5). Unexpectedly, inhibition of CPT1a with etomoxir (Figure 5A; p = 0.73; 100 μM) or by gene knockout (Figure 5C; p = 0.85) did not change G_KATP_ in α cells. Because β cells have a larger G_KATP_ (Briant et al., 2017), we used them to further interrogate any pharmacological effects of etomoxir on G_KATP_ (Figures 5E–5H). Etomoxir did not change G_KATP_ in β cells at low glucose (p = 0.96). We mimicked the effects of etomoxir on *[ATP]*_*i*_ by artificially decreasing *[ATP]*_*i*_. To this end, we continuously monitored G_KATP_ in standard whole cell while diluting the cell interior with a pipette solution containing 1 mM ATP (Figures 5I–5M). This would achieve a bona fide decrease in *[ATP]*_*i*_ because the ATP concentration in α cells at 1 mM glucose is >1 mM (Detimary et al., 1998). Decreasing *[ATP]*_*i*_ in this way did not change G_KATP_ in α cells (p = 0.93; Figures 5I and 5L), consistent with what has been shown in β cells (Tarasov et al., 2006). In a final set of experiments, we applied etomoxir in the presence of the K_ATP_ channel inhibitor tolbutamide (100 μM; Figure 5N). In these experiments, when the α cells were repolarized by injection of negative currents, etomoxir was still able to depolarize the membrane potential (p = 0.001; Figure 5O) and reduce AP amplitude (p = 0.042; Figure 5P). This effect of etomoxir must be independent of K_ATP_ channel closure. In conclusion, blockade of FAO via inhibition of CPT1 depolarizes the α cell membrane potential by a K_ATP_ channel-independent mechanism.

We therefore sought an ATP-dependent mechanism that could explain these data: namely, result in a depolarization of membrane potential, following a decrease in *[ATP]*_*i*_. It can be seen that there was an increase in the holding current (the current required to hold the membrane potential at −70 mV) following pharmacological inhibition of CTP1 (p = 0.008; Figure 5B), knockout of *Cpt1a* (p = 0.017; Figure 5D), or by artificially reducing *[ATP]*_*i*_ in 1 mM glucose (Figures 5J–5M; p = 0.049).

### FFA Oxidation Maintains α Cell Membrane Potential and Glucagon Secretion by Energizing the Na^+^-K^+^ Pump

The Na^+^-K^+^ pump is an ATPase, extruding intracellular Na^+^ in exchange for K^+^. Its operation is electrogenic and generates an outward current. Thus, reduced activity of the Na^+^-K^+^ pump would account for the increase in holding current following inhibition of CPT1a (Figures 5B and 5D). We therefore postulated that membrane potential and AP amplitude are maintained in low (1 mM) glucose because FAO energizes the Na^+^-K^+^ pump.

**Figure 6:**
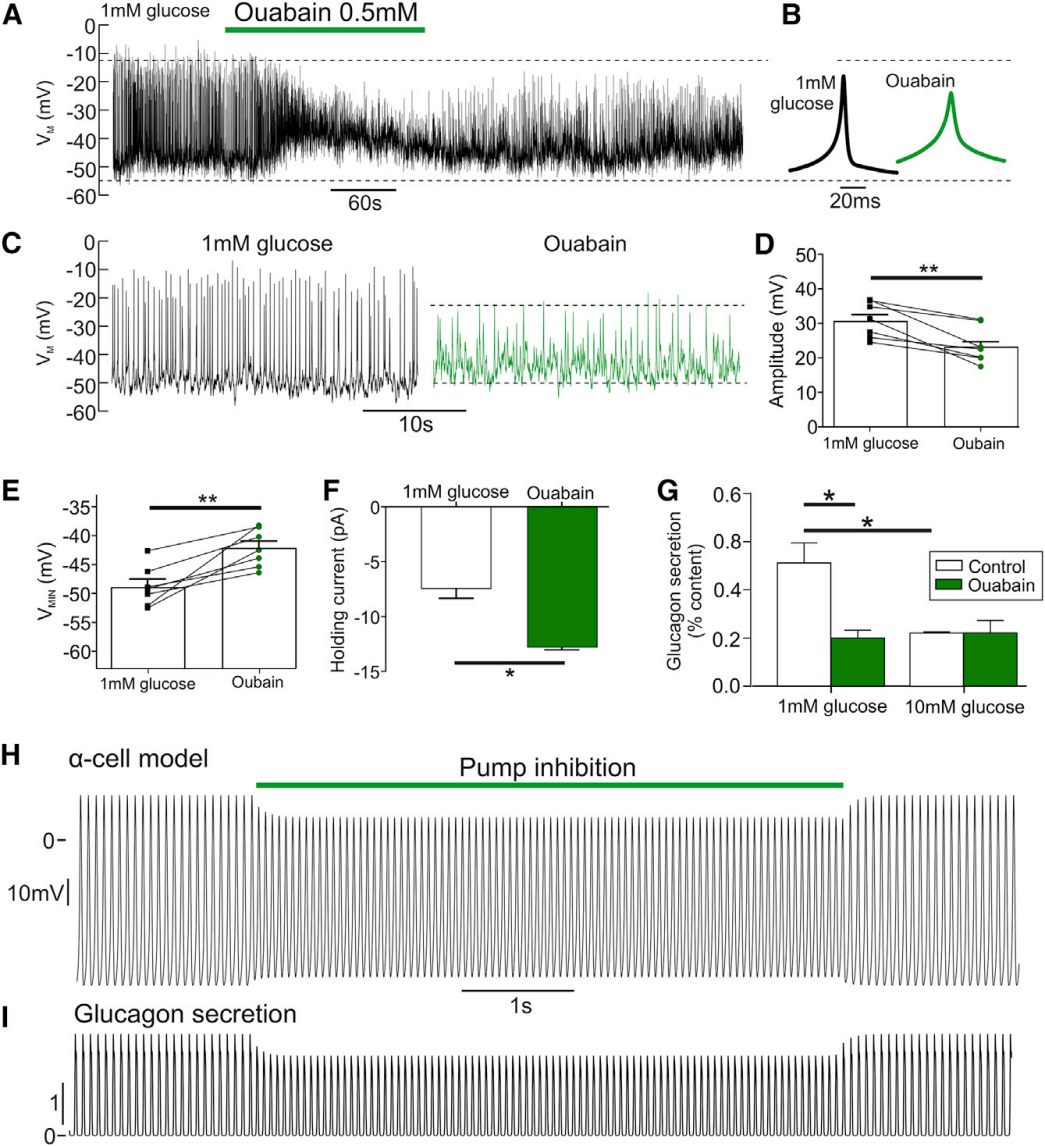
FFA Oxidation Maintains α Cell Membrane Potential by Energizing the Na^+^-K^+^ Pump (A) Electrical activity in a WT α cell in response to 1 mM glucose with or without ouabain (0.5 mM). (B) Average action potential waveform for (A) during 1 mM glucose and ouabain, measured over entire experimental condition. (C) Expanded view on 1 mM glucose and ouabain conditions for (A). (D) Grouped data for change in action potential amplitude in response to 1 mM glucose with or without ouabain (0.5 mM; 7 islets, 4 WT mice). (E) Grouped data for change in minimum membrane potential (V_MIN_) in WT α cells in response to 1 mM glucose with or without ouabain (0.5 mM; 7 islets, 4 WT mice). (F) The holding current in absolute value in WT α cells, in response to 1 mM glucose with or without ouabain (0.5 mM; 7 islets, 4 WT mice). (G) Glucagon secretion from WT islets at 1 and 10 mM glucose with or without ouabain (0.5 mM; n = 4 mice). (H) Mathematical model of α cell electrical activity demonstrates that a reduction of Na^+^-K^+^ pump activity (I_pump_) reduces action potential amplitude, mimicking blockade of CPT1a. (I) Accompanying model of glucagon secretion demonstrates that this results in a reduction of glucagon secretion, also mimicking blockade of CPT1a. All data are represented as mean ± SEM. Paired t test with Tukey post hoc or two-way ANOVA with Student-Newman-Keuls post hoc; ^∗^p < 0.05. See also Figure S1.

To investigate this, we exposed islets from WT mice to 0.5 mM ouabain in 1 mM glucose (Figure 6). Blockade of Na^+^-K^+^ pump activity decreased AP amplitude (p = 0.0005; Figures 6A–6D). Ouabain also reduced glucagon secretion in low glucose (p = 0.0015; Figure 6G), but did not change G_KATP_ in α cells (Figures S1D and S1E) or β cells (p = 0.45; Figures S1F and S1G). Thus, this depolarization was not due to a reduction in G_KATP_. Ouabain did, however, increase the magnitude of the holding current in both α cells (p = 0.0132; Figure 6F) and β cells (p = 0.0242; Figure S1H). Therefore, all of the effects of FAO inhibition on electrical activity (Figures 4 and 5) were mirrored by blockade of the Na^+^-K^+^ pump with ouabain. Together, these data suggest that Na^+^-K^+^ pump activity is high in low glucose, and preservation of this activity by FAO maintains a hyperpolarized membrane potential, and therefore AP amplitude and glucagon secretion, in α cells. To test the consistency of our experimental observations with this theory, we constructed a mathematical model of membrane potential in α cells (Figures 6H and 6I). Reducing energy supply to the Na^+^-K^+^ pump from FAO in the model recapitulated the experimental data, supporting our hypothesis.

### Reduced FAO in Human Islets Causes a Reduction in Electrical Activity and Glucagon Secretion

**Figure 7:**
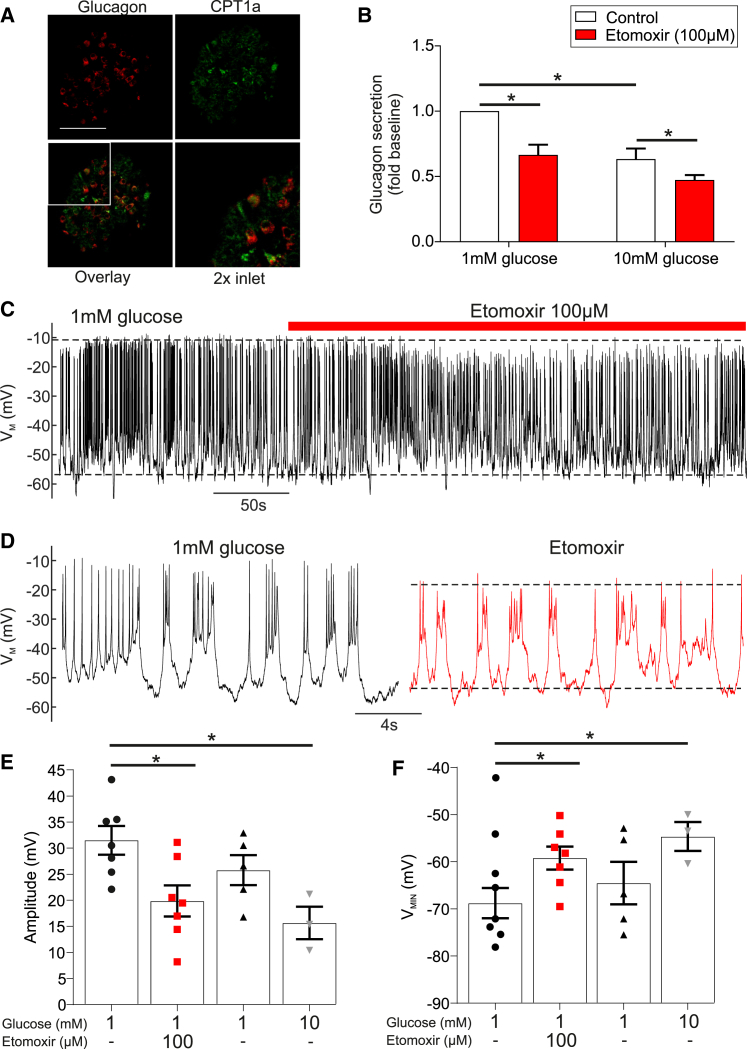
CPT1a Blockade in Human Islets Reduces α Cell Membrane Potential and Glucagon Secretion (A) Immunofluorescent detection of glucagon (red) and CPT1a (green) in isolated human islets (scale bar, 50 μm). (B) Glucagon secretion from human islets at 1 and 10 mM glucose with or without etomoxir (100 μM) reduced glucagon secretion (n = 3 donors). (C) Electrical activity in human α cells at 1 mM glucose with or without etomoxir (100 μM) (n = 3 donors, ≤7 cells). (D) Expanded view of 1 mM glucose and etomoxir conditions for (C). (E) Action potential amplitude in human α cells at 1 mM glucose with or without etomoxir (100 μM) and at 10 mM glucose (n = 3 donors, ≤7 cells). (F) Minimum potential in human α cells at 1 mM glucose with or without etomoxir (100 μM) and at 10 mM glucose (n = 3 donors, ≤7 cells). All data are represented as mean ± SEM. Paired t test with Tukey post hoc or two-way ANOVA with Student-Newman-Keuls post hoc; ^∗^p < 0.05.

Islets from human donors stained positive for CPT1a, with co-localization (∼90%) of glucagon and CPT1a (Figure 7A). Inhibition of FAO with etomoxir (100 μM) suppressed glucagon secretion at 1 mM glucose (p = 0.008; Figure 7B). Finally, analysis of membrane potential in α cells revealed that etomoxir reduces AP amplitude (p = 0.034; Figures 7C–7E) and depolarizes the membrane (p = 0.035; Figure 7F). These observations recapitulate our findings in mouse islets and suggest that FAO in human α cells contributes to the maintenance of membrane potential and glucagon secretion in low glucose conditions.

## Discussion

In this study we investigated the role of FAO in regulating glucagon secretion under hypoglycemic conditions. We found that pharmacological blockade or knockout of CPT1a profoundly reduced glucagon secretion from mouse islets at low glucose. These findings were mirrored in human islets with pharmacological inhibition of CPT1. In both mouse and human α cells, inhibition of CPT1 was associated with membrane potential depolarization and reduced AP amplitude.

Our findings suggest that during fasting, when blood glucose becomes low, fatty acids play a significant role in maintaining blood glucose by sustaining basal glucagon secretion. We show that FAO contributes to ATP production in low glucose conditions in α cells, and that this FFA-derived ATP maintains glucagon secretion by energizing the Na^+^-K^+^ pump. We propose that in low glucose concentrations, ATP generated by FAO is an essential energy supply for the Na^+^-K^+^ pump, keeping the α cell membrane sufficiently repolarized to prevent voltage-dependent inactivation of the ion channels involved in AP firing, thus allowing the generation of large-amplitude APs. When glucose becomes available, ATP derived from glucose oxidation triggers membrane depolarization by closure of K_ATP_ channels, leading to a K_ATP_-dependent reduction in glucagon secretion.

The K_ATP_ channel has been demonstrated to mediate the intrinsic regulation of glucagon secretion from α cells (MacDonald et al., 2007, Zhang et al., 2013). According to this model of counter-regulation, a reduction in glucose concentrations causes opening of K_ATP_ channels, a hyperpolarization of membrane potential, an increase in AP amplitude, and, therefore, glucagon secretion. However, this model is not wholly accepted (see Gylfe, 2013, Gylfe, 2016, Gylfe and Gilon, 2014). In low glucose, we observed that inhibition or reduction of FAO depolarized α cells and decreased glucagon secretion. If this depolarizing effect of inhibiting FAO were mediated by changes in K_ATP_ channel activity, we would expect to observe a reduction in K_ATP_ conductance. However, this is incompatible with our data because inhibition of FAO decreases *[ATP]*_*i*_, which would cause an increase in K_ATP_ conductance. Furthermore, we did not observe a change in K_ATP_ conductance in response to CPT1a inhibition, either pharmacologically or via knockout, suggesting that K_ATP_ channels are not directly involved.

To identify a possible mechanism underlying the effect on α cell electrical activity, we considered energy-consuming cellular mechanisms that result in membrane depolarization following a reduction in cellular ATP. The Na^+^-K^+^ pump is a major energy consumer in most types of cells (∼10% in muscle (Pirkmajer and Chibalin, 2016), ∼40% in neurons (Attwell and Laughlin, 2001), and ∼50% in the kidney (Clausen et al., 1991), utilizing more ATP than any other enzyme and consuming 19%–28% of whole-body ATP (Rolfe and Brown, 1997). The α cell appears to be no exception; we show that the application of the Na^+^-K^+^ pump inhibitor ouabain robustly depolarizes α cells. Considering that the Na^+^-K^+^ pump has a K_m_ of ∼0.4 mM for ATP (Javorková et al., 2009), α cell pump activity at 1 mM glucose would be drastically decreased when ATP levels are further reduced. Furthermore, this K_m_ is 30 times higher than the K_m_ of the K_ATP_ channel to ATP (Tarasov et al., 2006, Javorková et al., 2009). The pump would therefore turn off before the channel would be affected by the reduction in *[ATP]*_*i*_. We suggest that the reduction in ATP, which occurs when FAO is blocked with etomoxir or when *Cpt1a* is knocked out in α cells, leads to a reduction in Na^+^-K^+^ pump activity in low glucose.

Defects in mitochondrial β-oxidation have serious clinical consequences (Kompare and Rizzo, 2008) and account for a major cause of hypoglycemic seizures. There are at least 12 FAO disorders described, of which 10 are associated with routine fasting hypoglycemia (Grosse et al., 2006). CPT1a deficiency presents in infancy, is characterized by hypoketotic hypoglycemia (Ogier de Baulny et al., 1995, Greenberg et al., 2009), and is treated with frequent feedings to prevent prolonged fasting (Longo et al., 2006).

It has previously been suggested that the liver uses ATP generated from FAO to maintain glucose production (Staehr et al., 2003, Lam et al., 2003). However, recent findings suggest that hepatic FAO is expendable for maintaining 24-hr fasting blood glucose (Lee et al., 2016). Thus, loss of CPT1a activity in the liver of CPT1a-deficient patients may cause the hypoketonemia, but not the hypoglycemia. Our data support this and indicate that the hypoglycemia in these patients may be caused by a loss of FAO in the α cell and, consequently, reduced glucagon secretion.

## Experimental Procedures

All animal experiments were conducted in accordance with the UK Animals Scientific Procedures Act (1986) and University of Oxford ethical guidelines, and were approved by the local Ethical Committees. Human pancreatic islets were isolated (with ethical approval and clinical consent) at the Diabetes Research and Wellness Foundation Human Islet Isolation Facility (OCDEM, Oxford, UK) from pancreases of six non-diabetic donors. Donors were on average 61 years old (range 25–76) with a BMI of 24 (range 19.3–30) and HbA1c of 5.5% (range 5.3–5.9). Three of six donors were female.

### Animals and Generation of αCPT1a-KO Mice

C57BL/6j mice were used as WT mice in this study. To generate αCPT1a-KO, mice carrying a loxP insert flanking exons 11 and 12 of the *Cpt1a* gene (Schoors et al., 2015) were crossed with mice carrying Cre recombinase under the control of the proglucagon promoter (Parker et al., 2012). Mice homozygous for the loxP allele were used as controls and are referred to as such.

### *In Vivo* Measurements of Plasma Glucose, Glucagon, and Ketone Body Concentration

Plasma glucose, glucagon, and ketone body measurements were conducted *in vivo* on αCPT1a-KO and control mice in response to fasting. Mice were restrained, and a tail-vein sample of plasma was used to measure fed plasma glucose and ketone bodies. Mice were then individually caged for the 4-hr fasting period and given unrestricted access to water during this time. At the end of the fasting period, mice were restrained and a tail-vein sample of plasma was used to measure plasma glucose and ketone bodies. Mice were then culled by cervical dislocation and trunk plasma collected. The serum was then removed and stored at −80°C. Serum samples were used to measure plasma glucagon. Measurements were conducted using a dual mouse insulin/glucagon assay system (Meso Scale Discovery, MD, USA), according to the manufacturer’s protocol.

### Isolation of Pancreatic Islets

Mice at 12–16 weeks of age were killed by cervical dislocation (Schedule 1 procedure). Pancreatic islets were isolated by Liberase digestion followed by manual picking. Islets were used acutely and were, pending the experiments, maintained in tissue culture for <24 hr in RPMI 1640 (11879-020; GIBCO, Thermo Fisher Scientific) containing 1% penicillin/streptomycin (1214-122; GIBCO, Thermo Fisher Scientific), 10% fetal bovine serum (FBS; F7524-500G; Sigma-Aldrich), and 7.5 mM glucose before the measurements.

### Hormone Release Measurements

Measurements of insulin and glucagon secretion were performed using sequential incubations of isolated mouse and human islets as described in the Supplemental Information.

### Electrophysiology

All electrophysiological measurements were performed at 33°C to 34°C on α cells within intact islets (from αCPT1a-KO mice, littermate controls, WT C57BL/6j mice, and human islets). Membrane potential and whole-cell K_ATP_-current recordings were conducted using the perforated patch technique, as previously described (De Marinis et al., 2010). The composition of solutions is described in the Supplemental Information.

### ATP Imaging

The ATP/ADP sensor Perceval was used, as previously described (Adam et al., 2017).

### FFA Oxidation Measurements

αTC1-6 cells were cultured overnight in RPMI culture medium containing 5 mM glucose. On the day of the experiment, the cells were incubated in 0 mM glucose Krebs Ringer buffer (KRB) with or without etomoxir. The cells were then exposed to 0.3 mM palmitate containing 0.22 MBq [^3^H]palmitate for 1 hr. The supernatant was then subjected to a Falkes extraction and the aqueous phase assayed for H^3^ content. From this, β-oxidation was calculated and normalized to cell count. Further details are in the Supplemental Information.

### Mathematical Model of α Cell Membrane Potential

A mathematical model of Na^+^-K^+^ pump activity was added to a model of α cell membrane potential (Briant et al., 2018) and simulated in the simulation environment NEURON with a 25-μS time step. The model is further described in the Supplemental Information.

### Statistical Tests

All data are reported as mean ± SEM, unless otherwise stated. Statistical significance was defined as p < 0.05. All statistical tests were conducted in Prism (GraphPad Software, San Diego, CA, USA). For two groupings, a t test was conducted with the appropriate post hoc test. For more than two groupings, a one-way ANOVA was conducted. If there were two independent variables, a two-way ANOVA was conducted. If the data passed normality criteria (D’Agostino’s test of normality and Bartlett’s test of equal variances), a parametric test was conducted with the appropriate post hoc test (Tukey or Student-Neumann-Keuls). If the normality criteria were not met, a Kruskal-Wallis test with Dunn’s multiple comparison test was conducted.

## Supplemental Information

Document S1. Supplemental Experimental Procedures and Figures S1 and S2

Document S2. Article plus Supplemental Information
