# Supplementary material for: CPT1a-Dependent Long-Chain Fatty Acid Oxidation Contributes to Maintaining Glucagon Secretion from Pancreatic Islets
Source: Cell Rep. 2018 Jun 13;23(11):3300–11. doi: 10.1016/j.celrep.2018.05.035 (PMC6581793; doi:10.1016/j.celrep.2018.05.035)
Supplement: Document S1. Supplemental Experimental Procedures and Figures S1 and S2 [file mmc1.pdf]

Cell Reports, Volume *23*

Supplemental Information

CPT1a-Dependent Long-Chain Fatty Acid Oxidation Contributes to Maintaining Glucagon Secretion from Pancreatic Islets

Linford J.B. Briant, Michael S. Dodd, Margarita V. Chibalina, Nils J.G. Rorsman, Paul R.V. Johnson, Peter Carmeliet, Patrik Rorsman, and Jakob G. Knudsen

Figure S1

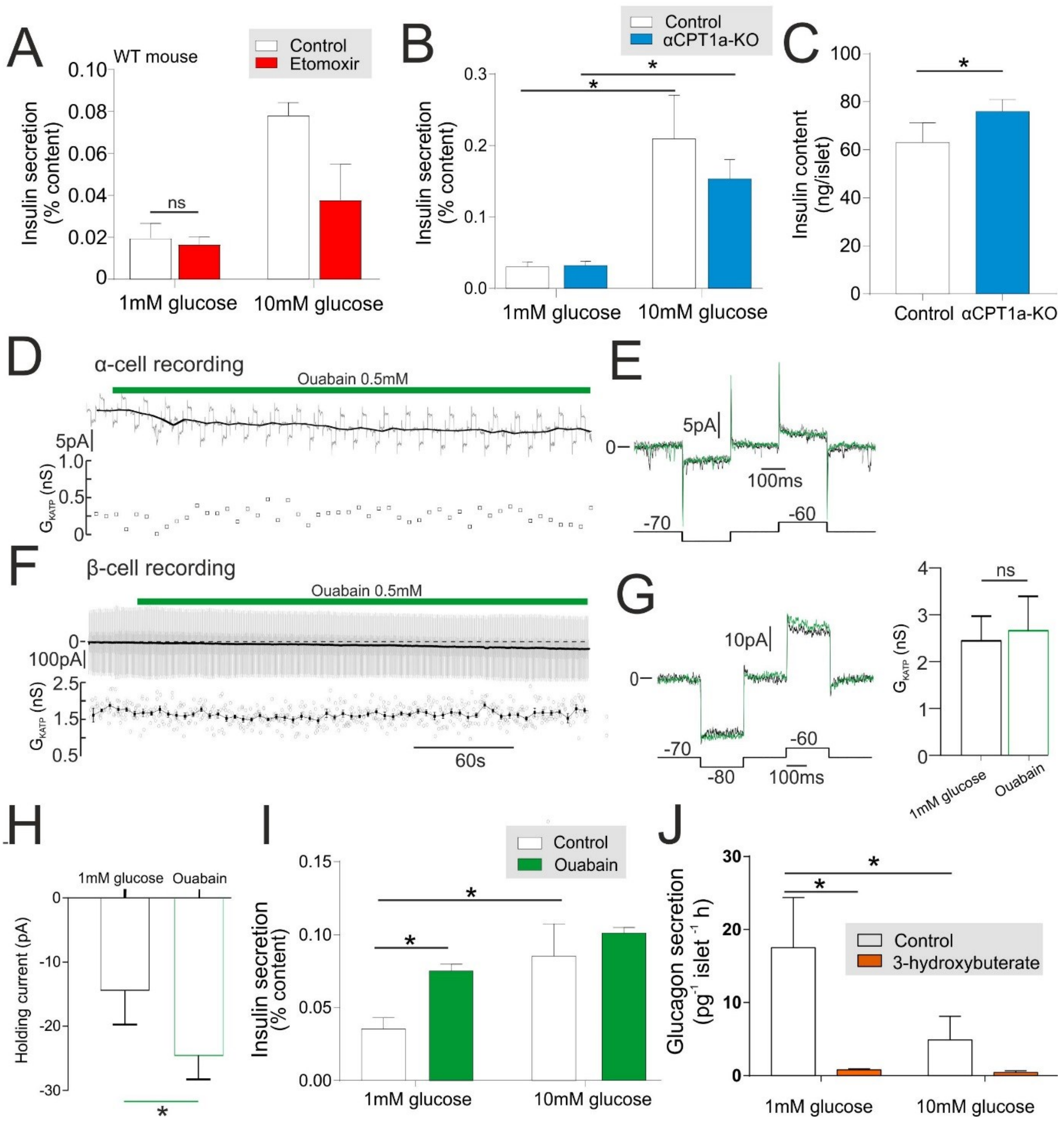

Supplementary Figure 1 (Related to Figure 1-3)

(A) Insulin secretion from WT islets at 1 and 10mM glucose with or without etomoxir (100 μM) reduced glucagon secretion (n=3).

(B) Insulin secretion from control and αCPT1a-KO mice at 1 and 10mM glucose (n=6).

(C) Insulin content from control and αCPT1a-KO islets (n=6).

(D) Continuously recording G_{KATP} in WT α-cells at 1mM glucose following ouabain (0.5mM) application.

(E) G_{KATP} in a WT α-cell at 1mM glucose with or without ouabain (0.5mM).

(F) Continuously recording G_{KATP} in a WT β-cell at 1mM glucose following ouabain (0.5mM) application

(G) G_{KATP} in a WT β-cell at 1mM glucose with or without ouabain

(I) Grouped G_{KATP} data from WT α-cell at 1mM glucose with or without ouabain (0.5mM) (3 cells from 3 mice).

(J) Glucagon secretion in WT mouse islets at 1 or 10mM glucose with or without 3-hydroxybuterate (0.5mM) (n=3).

All data are represented as mean ± SEM. Paired t-test with Tukey post-hoc or two-way ANOVA with Student Newman-Keuls post-hoc; * = $p<0.05$.

Figure S2

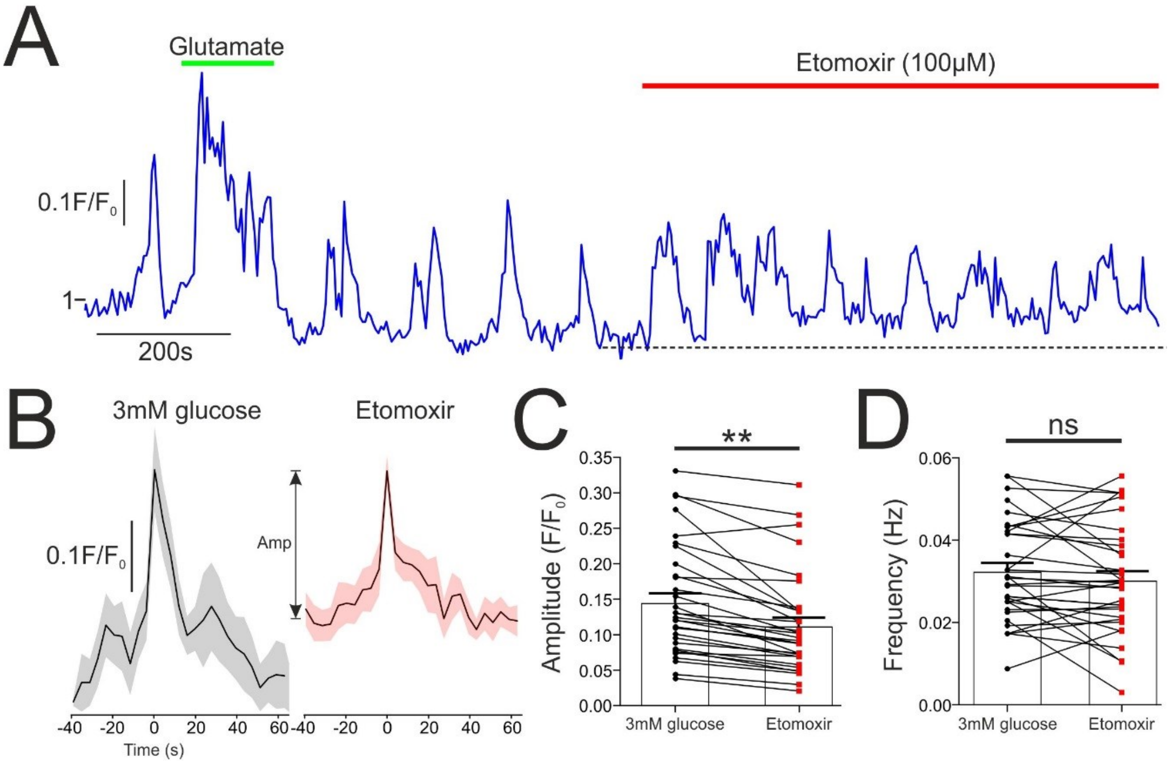

Supplementary Figure 2 (Related to Figure 4)

(A) Fluorescence intensity of intracellular calcium ($[Ca^{2+}]_i$) measured using Fluo-4 from intact pancreatic WT islets. Recordings were conducted in 3 mM glucose and 3 mM glucose with 100 µM etomoxir.

(B) For each experimental condition, the peak of each $[Ca^{2+}]_i$ spike was marked and a peak-triggered average waveform of $[Ca^{2+}]_i$ computed. The amplitude (Amp) of this average waveform was then measured.

(C) Amplitude of average $[Ca^{2+}]_i$ spike waveform (Amp) in 3 mM glucose with or without 100 µM etomoxir. (4 islets, 4 mice)

(D) Frequency of $[Ca^{2+}]_i$ spikes in 3 mM glucose with or without 100 µM etomoxir. (4 islets, 4 mice)

All data are represented as mean ± SEM. Paired t-test with Tukey post-hoc; ** = $p<0.01$.

Supplementary methods

Ethics
All animal experiments were conducted in accordance with the UK Animals Scientific Procedures Act (1986) and University of Oxford ethical guidelines, and were approved by the local Ethical Committees. Human pancreatic islets were isolated, with ethical approval and clinical consent, at the Diabetes Research and Wellness Foundation Human Islet Isolation Facility (OCDEM, Oxford, UK).

Animals
All animals were kept in a specific pathogen-free (SPF) facility under a 12:12 hour light:dark cycle at 22 °C, with unrestricted access to standard rodent chow and water. C57BL/6j mice used in this study are referred to as wild-type (WT) mice. To generate α-cell specific *Cpt1a* knockout mice (αCPT1a-KO), mice carrying a loxP insert flanking exons 11 and 12 of the *Cpt1a* gene (Schoors et al., 2015) were crossed with mice carrying a Cre recombinase under the control of the proglucagon promoter (Parker et al., 2012). Mice homozygous for the loxP allele were used as controls, and are referred to as such.

***In vivo* measurements of plasma glucose, glucagon and ketone body concentration**
Plasma glucose, glucagon and ketone body measurements were conducted *in vivo* on αCPT1a-KO and control mice in response to fasting. Experiments followed a strict time schedule to minimise experimental variation. Plasma glucose was measured with an Accu-Chek Aviva (Roche Diagnostic, UK) and plasma ketone bodies with a FreeStyle Libre system (Abbott Laboratories Ltd, Maidenhead, UK). Bovine aprotinin 2µl (4 TIU/ml) (Sigma-Aldrich, UK) was added to all plasma samples.
Fed plasma glucose: Mice were restrained and a tail vein sample of blood was used to measure fed plasma glucose and ketone bodies. At this stage, some mice were culled, and the liver was removed, snap frozen in liquid nitrogen and stored at -80 °C. Other mice were not culled, but instead used for subsequent *in vivo* studies.
Fasting studies: Mice were restrained and fed plasma glucose was measured as above. Mice were then individually caged for the 4 hour fasting period (8.30am-12.30pm) and given unrestricted access to water during this time. At the end of the fasting period, mice were restrained and a tail vein sample of plasma was used to measure plasma glucose and ketone bodies. Mice were then culled by cervical dislocation and trunk plasma collected in EDTA coated tubes. Blood sampels were kept on ice and imediately centrifuged at 2700 rpm for 10 min to obtain plasma. The plasma was then removed and stored at -80 °C.
Plasma glucagon measurements: Plasma samples (stored at -80 °C prior) were used to measure plasma glucagon. Measurements were conducted using a mouse glucagon assay system (Mercodia, Upsala, Sweden), according to the manufacturers protocol.

Pharmacological blockade of CPT1

Pharmacological blockade of CPT1 was achieved using the CPT1 inhibitor etomoxir (Sigma-Aldrich, UK). Concentrations of 25-200 µM have been used to block CPT1 activity in islet cells in other studies (Chen et al., 1994, Lehtihet et al., 2003). Prolonged exposure to high concentrations (1 mM) of etomoxir is known to increase ROS, deplete ATP and lead to cell death (Pike et al., 2011). Although our application of etomoxir was short (<1 hour), we opted for 100 µM to avoid this. This concentration of 100 µM was used throughout this study.

Isolation of pancreatic islets
Mice at 12-16 weeks of age were killed by cervical dislocation (Schedule 1 procedure). Pancreatic islets were isolated by liberase digestion followed by manual picking. Islets were used acutely and were, pending the experiments, maintained in tissue culture for <24 hour in RPMI 1640 (11879-020, Gibco, Thermo Fisher Scientific) containing 1% pennicillin/streptomycin (1214-122, Gibco, Thermo Fisher Scientific), 10%FBS (F7524-500G, Sigma-Aldrich) and 7.5mM glucose prior to the measurements.

Cell culture experiments
The αTC1-clone 6 cell line (αTC1-6; CRL-2934, ATCC), was cultured in RPMI 1640 (118279-020, Gibco, Thermo Fisher Scientific) containing 15mM Hepes (15630-56, Gibco, Thermo Fisher Scientific), 1% penicillin/streptomycin (15140-122, Gibco, Thermo Fisher Scientific), 10%FBS (10270-10, Gibco, Thermo Fisher Scientific) and 15mM glucose. For all experiments, cells were plated in 12 or 6 well plates, and, after 3 days of culture, glucose concentration was lowered to 5 mM over night and experiments were performed the next day. For knock down of *Cpt1a*, αTC1-6 cells were cultured for 2 days in standard medium and then

transfected with scrambled control siRNA (SR30004,Origene) or siRNA specifically targeting *Cpt1a* mRNA (J-042456-10-0002, Dharmacon Inc.). After 36 hour, medium was changed to culture medium containing 5 mM glucose and experiments were performed the next day. The efficiency of downregulation was assessed by Western blot.

Evaluation of protein expression
For evaluation of protein expression, αTC1-6 cells were lysed in Hepes KOH pH7.6 buffer containing 0.1% TritonX-100 (Sigma-Aldrich) and protease and phosphatase inhibitors (Roche, UK). Lysates were immedaitely sonicated and then stored at -80°C for further analysis. Protein content was determined by bicinchoninic acid assay (Thermo Fisher Scientific, UK).

Patch-clamp electrophysiology in islets
Mouse and human islets were used for patch-clamp electrophysiological recordings. These recordings (in intact islets) were performed at 33-34 ^{0}C using an EPC-10 patch-clamp amplifier and PatchMaster software (HEKA Electronics, Lambrecht/Pfalz, Germany). Currents were filtered at 2.9 kHz and digitized at > 10 kHz. A new islet was used for each recording. Membrane potential (V_M) recordings were conducted using the perforated patch-clamp technique, as previously described (De Marinis et al., 2010, Zhang et al., 2013). The pipette solution contained (in mM) 76 K_2SO_4, 10 NaCl, 10 KCl, 1 $MgCl_2{\cdot}6H_20$ and 5 Hepes (pH 7.35 with KOH). For these experiments, the bath solution contained (mM) 140 NaCl, 3.6 KCl, 10 Hepes, 0.5 $MgCl_2{\cdot}6H20$, 0.5 $Na_2H_2PO_4$, 5 $NaHCO_3$ and 1.5 $CaCl_2$ (pH 7.4 with NaOH). Amphotericin B (final concentration of 25mg/mL, Sigma-Aldrich) was added to the pipette solution to give electrical access to the cells (series resistance of <100 MΩ). α-cells were confirmed by their activity at 1 mM glucose and a logistic regression model that can identify cell type with 94% accuracy (Briant et al., 2017). Recordings were made during brief (5-10 min) exposure to etomoxir (100 μM), and action potential morphology was quantified and compared.

The morphology of action potentials and frequency of firing was calculated in MATLAB v. 6.1 (2000; The MathWorks, Natick, MA). In brief, a peak-find algorithm was used to detect action potentials. This was then used to calculate firing frequency in different experimental conditions. The peaks were then used as triggers to create a waveform-triggered average of membrane potential in different experimental conditions. The window chosen for this analysis was the entire experimental condition. This average action potential waveform was generated over a symmetric 50 ms window (25 ms before and after the trigger). The minimal membrane potential (V_{MIN}) was defined as the minimal membrane potential preceeding the action potential in this window. The action potential amplitude was defined as the amplitude of this average waveform. Analysis of action potential morphology in αCPT1a-KO and control mice was conducted 'blind' to mouse strain (blinding was conducted by an acknowledged independent researcher).

Quantitive imaging of ATP in islets
Time-lapse imaging of the ATP/ADP ratio in WT mouse islets was performed using 14× magnification on a Zeiss AxioZoom V16 microscope, as previously described (Adam et al., 2017). In brief, islets were transduced with an adenovirus delivering Perceval, a recombinant sensor of ATP/ADP. Groups of islets were imaged simultaneously 24 hour post-transduction at glucose concentrations as indicated, with single-cell resolution. Time-lapse images were collected every 30 s, and the bath solution was perifused at 60 μl/min at 34 °C. The bath solution was as described for patch-clamp electrophysiology.

Ca^{2+} imaging in islets

Time-lapse imaging of the intracellular Ca^{2+} concentration ($[Ca^{2+}]_i$) in WT mouse islets was performed on the inverted Zeiss AxioVert 200 microscope, equipped with the Zeiss LSM 510-META laser confocal scanning system, using a 40x/1.3 NA objective. Mouse islets were loaded with 6 mM of the Ca^{2+}-sensitive dye Fluo-4 for 90 min before being transfered to a recording chamber. Mice were then continuously perfused with bath solution (same solution as described for patch-clamp electrophysiology, above) at a rate of 200 μL/min. Fluo-4 was excited at 488 nm and fluorscence emission collected at 530 nm. The pinhole diameter was kept constant, and frames of 256x256 pixels were taken every 1-3 s. α-cells were identified by the presence of oscillations in $[Ca^{2+}]_i$ in low (3 mM) glucose and an excitatory response to glutamate.

Hormone secretion measurements
Islets: Islets isolated from WT, αCPT1a-KO and control mice were incubated for 1h in RPMI supplemented with 7.5 mM glucose in a cell culture incubator. Size-matched batches of 20 islets were pre-incubated in 0.2 ml

KRB with 2mg/ml BSA (S6003, Sigma-Aldrich) and 3 mM glucose for 1 hour in a water-bath at 37 ^{0}C. Following this islets were sequentially subjected to 0.2 ml KRB with 2 mg/ml BSA with 1 mM or 10 mM glucose for 1 hour. For Ketone body experiments islets were subjected to with 1 mM or 10 mM glucose with or without 0.5mM 3-hydroxybuterate for 1 hour After each incubation, the supernatant was removed, quickly frozen and stored at -80 ^{0}C. At the end of the experiment the islets were lysed in 0.1 ml of HCl:ethanol (1:15) and samples were sonicated and stored at -80 ^{0}C.
αTC1-6 cells: on the day of the experiment, cells were pre-incubated for 1 hour at 0 mM glucose KRB, with or without etomoxir (100 μM). The cells were then incubated for 1 hour in KRB containing either 1 or 10 mM glucose. After 1 hour, the supernatant was removed and centrifuged at 800 g, 4 °C for 10 min, to remove dead cell debris. The supernatant was transferred to clean eppendorf tubes and stored at -80 °C. Cells in the plate were lysed with 0.5 ml of HCl:ethanol (1:15), sonicated and stored at -80°C. Glucagon and insulin from secretion and content samples were measured using MSD mouse insulin/glucagon duplex sandwich ELISA (Mesoscale Discovery, USA), according to the manufacturers protocol.

Pancreas perfusion: Measurements of glucagon secretion was performed using *in situ* pancreatic perfusion. Briefly, the aorta was ligated above the coeliac artery and below the superior mesenteric artery and then cannulated. The pancreas was perfused with KRB containing varying concentrations of glucose in the following order 4, 6 and then 10 mM glucose at a speed of 0.240 ml/min using an Ismatec Reglo Digital MS2/12 peristaltic pump. The perfusate was maintained at 37°C using a Warner Instruments temperature control unit TC-32 4B in conjunction with a tube heater (Warner Instruments P/N 64-0102) and a Harvard Apparatus heated rodent operating table. The effluent was collected in intervals of 2 min in to 96 well plate on ice containing aprotenin. Samples were subsequently stored at -80°C. Glucagon content in perfusate were measured using mouse glucagon RIA (Euro-diagnostica, Sweden), according to the manufacturers protocol.

FFA oxidation measurements
αTC1-6 cells were cultured over night in RPMI culture medium containing 5 mM glucose. On the day of the experiment, the cells were incubated in KRB with 0 mM glucose and the relevant treatment (e.g. etomoxir). The cells were then exposed to 0.3 mM palmitate containing 0.22Mbeq H^3palmitate for 1 hour. The supernatant was then subjected to a Falkes extraction and the aquaeus phase assayed for H^3 content. From this, β-oxidation was calculated and normalised to cell count.

Immunohistochemistry and immunoblotting
Mouse or human pancreata or islets were fixed in 0.4% PFA 10% neutral-buffered formalin, dehydrated and processed for paraffin wax embedding and sectioning (3μm).

Immunostaining was performed using antibodies to CPT1a (ab128568, ABCAM, Cambridge, UK), CPT1b (ABCAM, Cambridge, UK), glucagon (ABCAM, Cambridge, UK) or glucagon (Sigma-Aldrich). CPT1b and glucagon staining was visualised using anti rabbit Alexafluor 488 (Molecular Probes, Thermo Fisher Scientific) or anti rabbit FITC secendary antibody. CPT1a staining was enhaced using VectaFluor™ Excel Amplified DyLight® 594 Anti-Mouse IgG Kit (DK-2594, Vectorlabs). Images were acquired with a Zeiss LSM510 META confocal imaging system.

For immunoblotting, cell lysates were diluted to a concentration of 1μg/ml in 2x Sample Buffer (BioRad) with β-mercaptoethanol (Sigma-Aldrich). After addition of the Laemmli sample buffer the proteins were separated usign SDS-PAGE, transferred to a PVDF membranes and visualised using antibodies to CPT1a, G6PC, β-actin (AbCam, UK), PEPCK (Cayman Chemical), calnexin (Millipore Merk) and HRP-conjugated secondary antibodies (Thermo Fisher Scientific, Rugby, UK). Blots were developed using ECL detection reagent (BioRad), images acquired on a ChemiDoc imager (Bio-Rad) and analysed using ImageLab software (BioRad). All data were normalised to either calnexin or β-actin and expressed as fold change of control.

Mathematical model of α-cell membrane potential
All simulations were conducted in the simulation environment NEURON using CVODE and a 25 μs timestep (Carnevale and Hines, 2006). The equation describing membrane potential (V_M) in the α-cell model is:

$$C_{cell}\frac{dV_M}{dt} = -(I_{CaL} + I_{CaN} + I_{CaT} + I_{Na} + I_K + I_{KATP} + I_{KA} + I_L) \quad (1)$$

where: C_{cell} is the cell capacitance; I_{CaL}, I_{CaN} and I_{CaT} are L-, N-, and T-type voltage-dependent Ca^{2+} currents, respectively; I_{Na} is a voltage-dependent Na^+ current; I_K is a delayed rectifier K^+ current; I_{KA} is an A-type

voltage-dependent K$^+$ current; $I_{K(ATP)}$ is an ATP-sensitive K$^+$ current; I_L is a leak current. This model is described in detail in (Briant et al., 2018). In addition, we modelled the current due to the Na$^+$-K$^+$ pump (Na$^+$/K$^+$-ATPase), I_{pump}. The equations describing this current are

$$I_{pump} = \frac{\overline{I_{pump}}}{1 + \left({k_m}/{[Na^+]_i}\right)^n}$$

as described in Canavier (1999). This current writes to the transmembrane Na$^+$ and K$^+$ currents in the NEURON environent, as follows:

$$I_{Na} = 3 \cdot I_{pump}$$
$$I_K = -2 \cdot I_{pump}$$

To mimic reduced ATP supply to the Na$^+$-K$^+$ pump, we reduced the maximal pump current, $\overline{I_{pump}}$.

Glucagon release was modelled by using the 4 variable system described by Yamada and Zucker (1992) and later simplified by Destexhe et al. (1994). In brief, the intracellular calcium concentration $[Ca^{2+}]$ was modelled as the sum of calcium fluxes due to the total calcium current ($I_{Ca} = I_{CaL} + I_{CaN} + I_{CaT}$) and a calcium buffering term:

$$\frac{d[Ca^{2+}]}{dt} = \frac{2I_{Ca}}{F_d \cdot d} + \frac{([Ca^{2+}]_0 - [Ca^{2+}])}{\tau} \quad (2)$$

Here, calcium is buffered to $[Ca^{2+}]_0$ with time-constant τ, F_d is Faradays constant and d is the depth of the calcium domain. This calcium concentration drives a system of differential equations describing glucagon vesicle dynamics:

$$\frac{d[F_A]}{dt} = k_b([F_{max}] - [F_A] - [V_A])[Ca^{2+}]^4 - k_u[F_A] - k_1[F_A][V] + k_2[V_A] \quad (3)$$

$$\frac{d[V_A]}{dt} = k_1[F_A][V] - (k_2 + k_3)[V_A] \quad (4)$$

$$\frac{d[GCG]}{dt} = Nk_3[V_A] - k_h[GCG] \quad (5)$$

Here, calcium ions are assumed to reversibly bind to a fusion protein F. Four calcium ions bind to this protein at a rate k_b, activating it. The concentration of activiated fusion protein is $[F_A]$, coming from a pool of inactivated proteins with concentration $[F_{max}]$. The reverse process has an unbinding rate k_u. An activated fusion protein binds to a vesicle (V) at a rate k_1, activated it (V_A). This process is reversible with unbind rate k_2. The concentrations of inactivated and activated vesicles are $[V]$ and $[V_A]$, respectively. Destexhe et al. (1994) simplified this system by assuming that there exists an inexhaustible pool of inactivated vesicles, ready for activation. In particular, $[V]$ is constant and not depleted. This assumption is adopted. An activated vesicle is then able to fuse to the membrane of the cell, and release its contents. An activated vesicle releases N molecules of glucagon (GCG) at a rate k_3. The concentration of glucagon released is $[GCG]$. This is depleted in the extracellular space by diffusion, degradation and reuptake at a rate k_h.

Statistical tests

All data are reported as mean ± S.E.M., unless otherwise stated. Statistical significance was defined as $p<0.05$. All statistical tests were conducted in Prism5 (GraphPad Software, San Diego, CA). For two groupings, a *t*-test was conducted. If the data were non-parametric, a Mann-Whitney test was conducted. For more than two groupings, a one-way ANOVA was conducted. If there were two independent variables, a two-way ANOVA was conducted. If the data passed normality criteria (D'Agostino's test of normality and Bartlett's test of equal variances), a parametric test was conducted with the appropriate post hoc test (Tukey or Student Neumann Keuls). If the normality criteria were not met, a Kruskal–Wallis test with Dunn's multiple comparison test was conducted.

References

ADAM, J., RAMRACHEYA, R., CHIBALINA, M. V., TERNETTE, N., HAMILTON, A., TARASOV, A. I., ZHANG, Q., REBELATO, E., RORSMAN, N. J. G., MARTIN-DEL-RIO, R., LEWIS, A., OZKAN, G., DO, H. W., SPEGEL, P., SAITOH, K.,

KATO, K., IGARASHI, K., KESSLER, B. M., PUGH, C. W., TAMARIT-RODRIGUEZ, J., MULDER, H., CLARK, A., FRIZZELL, N., SOGA, T., ASHCROFT, F. M., SILVER, A., POLLARD, P. J. & RORSMAN, P. 2017. Fumarate Hydratase Deletion in Pancreatic beta Cells Leads to Progressive Diabetes. *Cell Rep,* 20**,** 3135-3148.

BRIANT, L. J., ZHANG, Q., VERGARI, E., KELLARD, J. A., RODRIGUEZ, B., ASHCROFT, F. M. & RORSMAN, P. 2017. Functional identification of islet cell types by electrophysiological fingerprinting. *J R Soc Interface,* 14.

BRIANT, L. J. B., REINBOTHE, T. M., SPILIOTIS, I., MIRANDA, C., RODRIGUEZ, B. & RORSMAN, P. 2018. delta-cells and beta-cells are electrically coupled and regulate alpha-cell activity via somatostatin. *J Physiol,* 596**,** 197-215.

CANAVIER, C. C. 1999. Sodium dynamics underlying burst firing and putative mechanisms for the regulation of the firing pattern in midbrain dopamine neurons: a computational approach. *J Comput Neurosci,* 6**,** 49-69.

CARNEVALE, N. T. & HINES, M. L. 2006. *The NEURON book,* Cambridge, UK ; New York, Cambridge University Press.

CHEN, S., OGAWA, A., OHNEDA, M., UNGER, R. H., FOSTER, D. W. & MCGARRY, J. D. 1994. More direct evidence for a malonyl-CoA-carnitine palmitoyltransferase I interaction as a key event in pancreatic beta-cell signaling. *Diabetes,* 43**,** 878-83.

DE MARINIS, Y. Z., SALEHI, A., WARD, C. E., ZHANG, Q., ABDULKADER, F., BENGTSSON, M., BRAHA, O., BRAUN, M., RAMRACHEYA, R., AMISTEN, S., HABIB, A. M., MORITOH, Y., ZHANG, E., REIMANN, F., ROSENGREN, A. H., SHIBASAKI, T., GRIBBLE, F., RENSTROM, E., SEINO, S., ELIASSON, L. & RORSMAN, P. 2010. GLP-1 inhibits and adrenaline stimulates glucagon release by differential modulation of N- and L-type Ca2+ channel-dependent exocytosis. *Cell Metab,* 11**,** 543-53.

DESTEXHE, A., MAINEN, Z. F. & SEJNOWSKI, T. J. 1994. Synthesis of models for excitable membranes, synaptic transmission and neuromodulation using a common kinetic formalism. *J Comput Neurosci,* 1**,** 195-230.

LEHTIHET, M., WELSH, N., BERGGREN, P. O., COOK, G. A. & SJOHOLM, A. 2003. Glibenclamide inhibits islet carnitine palmitoyltransferase 1 activity, leading to PKC-dependent insulin exocytosis. *Am J Physiol Endocrinol Metab,* 285**,** E438-46.

PARKER, H. E., ADRIAENSSENS, A., ROGERS, G., RICHARDS, P., KOEPSELL, H., REIMANN, F. & GRIBBLE, F. M. 2012. Predominant role of active versus facilitative glucose transport for glucagon-like peptide-1 secretion. *Diabetologia,* 55**,** 2445-2455.

PIKE, L. S., SMIFT, A. L., CROTEAU, N. J., FERRICK, D. A. & WU, M. 2011. Inhibition of fatty acid oxidation by etomoxir impairs NADPH production and increases reactive oxygen species resulting in ATP depletion and cell death in human glioblastoma cells. *Biochim Biophys Acta,* 1807**,** 726-34.

SCHOORS, S., BRUNING, U., MISSIAEN, R., QUEIROZ, K. C. S., BORGERS, G., ELIA, I., ZECCHIN, A., CANTELMO, A. R., CHRISTEN, S., GOVEIA, J., HEGGERMONT, W., GODDE, L., VINCKIER, S., VAN VELDHOVEN, P. P., EELEN, G., SCHOONJANS, L., GERHARDT, H., DEWERCHIN, M., BAES, M., DE BOCK, K., GHESQUIERE, B., LUNT, S. Y., FENDT, S.-M. & CARMELIET, P. 2015. Fatty acid carbon is essential for dNTP synthesis in endothelial cells. *Nature,* 520**,** 192-197.

YAMADA, W. M. & ZUCKER, R. S. 1992. Time course of transmitter release calculated from simulations of a calcium diffusion model. *Biophys J,* 61**,** 671-82.

ZHANG, Q., RAMRACHEYA, R., LAHMANN, C., TARASOV, A., BENGTSSON, M., BRAHA, O., BRAUN, M., BRERETON, M., COLLINS, S., GALVANOVSKIS, J., GONZALEZ, A., GROSCHNER, L. N., RORSMAN, N. J., SALEHI, A., TRAVERS, M. E., WALKER, J. N., GLOYN, A. L., GRIBBLE, F., JOHNSON, P. R., REIMANN, F., ASHCROFT, F. M. & RORSMAN, P. 2013. Role of KATP channels in glucose-regulated glucagon secretion and impaired counterregulation in type 2 diabetes. *Cell Metab,* 18**,** 871-82.
