# Supplementary material for: CPT1a-Dependent Long-Chain Fatty Acid Oxidation Contributes to Maintaining Glucagon Secretion from Pancreatic Islets
Source: Cell Rep. 2018 Jun 13;23(11):3300–11. doi: 10.1016/j.celrep.2018.05.035 (PMC6581793; doi:10.1016/j.celrep.2018.05.035)
Supplement: Document S2. Article plus Supplemental Information [file mmc2.pdf]

Cell Reports

Article

CPT1a-Dependent Long-Chain Fatty Acid Oxidation Contributes to Maintaining Glucagon Secretion from Pancreatic Islets

Graphical Abstract

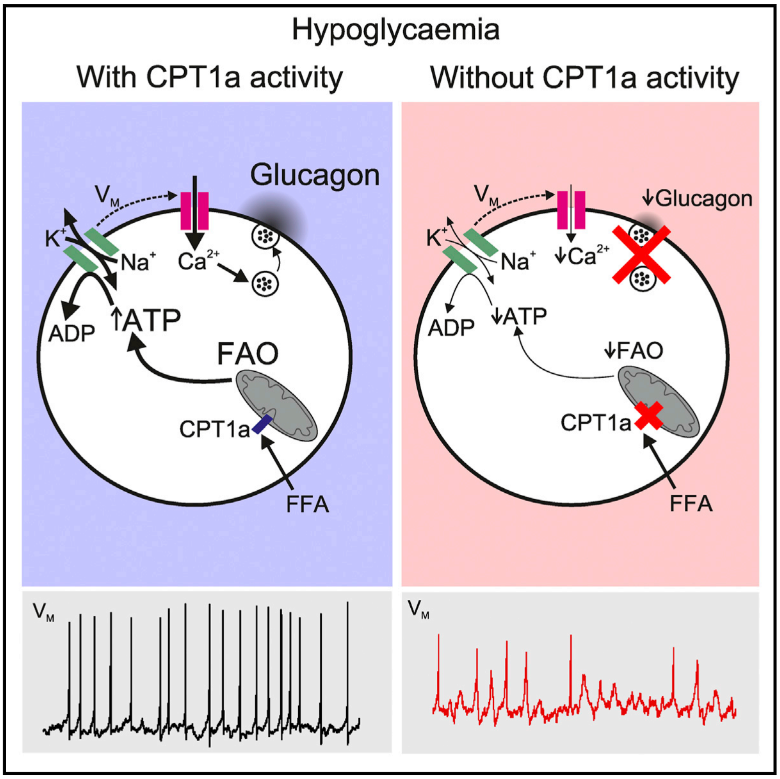

Highlights

- Glucagon secretion in low glucose is maintained by CPT1a-dependent FAO
- Loss of CPT1a-dependent FAO in mouse and human islets decreases glucagon secretion
- CPT1a-dependent FAO maintains glucagon secretion by supplying ATP to the Na^+-K^+-ATPase
- CPT1a-dependent FAO contributes to the counter-regulatory secretion of glucagon

Authors

Linford J.B. Briant, Michael S. Dodd, Margarita V. Chibalina, ..., Peter Carmeliet, Patrik Rorsman, Jakob G. Knudsen

Correspondence

jakob.knudsen@ocdem.ox.ac.uk

In Brief

Glucagon is secreted from pancreatic α cells in hypoglycemic conditions. Briant et al. demonstrate that this response is fueled by fatty acid oxidation. The energy generated by oxidation is used to maintain membrane potential dynamics, action potential morphology, and Na^+-K^+ pump activity.

Briant et al., 2018, Cell Reports *23*, 3300–3311
June 12, 2018 © 2018 The Author(s).
https://doi.org/10.1016/j.celrep.2018.05.035

CellPress

OPEN ACCESS
CellPress

Cell Reports
Article

CPT1a-Dependent Long-Chain Fatty Acid Oxidation Contributes to Maintaining Glucagon Secretion from Pancreatic Islets

Linford J.B. Briant,[1,2] Michael S. Dodd,[3,4] Margarita V. Chibalina,[1] Nils J.G. Rorsman,[1] Paul R.V. Johnson,[1,5] Peter Carmeliet,[6] Patrik Rorsman,[1,7] and Jakob G. Knudsen[1,8,*]
[1]Oxford Centre for Diabetes, Endocrinology and Metabolism, Radcliffe Department of Medicine, University of Oxford, Oxford OX3 7LE, UK
[2]Department of Computer Science, University of Oxford, Oxford OX1 3QD, UK
[3]Department of Physiology, Anatomy & Genetics, University of Oxford, Parks Road, Oxford OX1 3PT, UK
[4]Faculty of Health and Life Sciences, Coventry University, Coventry CV1 5FB, UK
[5]Oxford National Institute for Health Research, Biomedical Research Centre, Churchill Hospital, Oxford OX3 7LJ, UK
[6]Laboratory of Angiogenesis and Vascular Metabolism, VIB-KU Leuven Center for Cancer Biology, Leuven, Belgium
[7]Metabolic Research, Department of Neuroscience and Physiology, Sahlgrenska Academy, University of Göteborg, Box 433, 405 30 Göteborg, Sweden
[8]Lead Contact
*Correspondence: jakob.knudsen@ocdem.ox.ac.uk
https://doi.org/10.1016/j.celrep.2018.05.035

SUMMARY

Glucagon, the principal hyperglycemic hormone, is secreted from pancreatic islet α cells as part of the counter-regulatory response to hypoglycemia. Hence, secretory output from α cells is under high demand in conditions of low glucose supply. Many tissues oxidize fat as an alternate energy substrate. Here, we show that glucagon secretion in low glucose conditions is maintained by fatty acid metabolism in both mouse and human islets, and that inhibiting this metabolic pathway profoundly decreases glucagon output by depolarizing α cell membrane potential and decreasing action potential amplitude. We demonstrate, by using experimental and computational approaches, that this is not mediated by the K_{ATP} channel, but instead due to reduced operation of the Na^+-K^+ pump. These data suggest that counter-regulatory secretion of glucagon is driven by fatty acid metabolism, and that the Na^+-K^+ pump is an important ATP-dependent regulator of α cell function.

INTRODUCTION

Type 2 diabetes mellitus (T2DM) is a metabolic disorder characterized by hyperglycemia, insulin resistance, and insufficient insulin secretion from islet β cells (Leahy, 2005). However, it is becoming increasingly apparent that over-secretion of glucagon from pancreatic α cells also contributes to the increased hepatic glucose production and associated hyperglycemia in T2DM. The abnormalities in glucagon secretion in T2DM include both loss of adequate suppression under hyperglycemic conditions (Cryer, 2002, 2008; Cryer et al., 2003; Dunning et al., 2005; D'Alessio, 2011; Unger and Cherrington, 2012) and insufficient release during episodes of hypoglycemia (Gerich et al., 1973; Bolli and Perriello, 1990; Shamoon et al., 1994).

Despite the centrality of glucagon in the etiology of T2DM, the mechanisms by which glucagon secretion is regulated at low glucose have not been fully elucidated. Several paracrine processes have been demonstrated to alter glucagon secretion (Gromada et al., 2007; Gylfe, 2013, 2016; Caicedo, 2013; Gylfe and Gilon, 2014). However, in low glucose, glucagon secretion is controlled by mechanisms intrinsic to the α cell (Vieira et al., 2007; Zhang et al., 2013).

The β cell is tailored to deal with conditions of high glucose: the high-K_m GLUT transporter (GLUT2), high-K_m glucokinase, and high respiratory capacity in β cells result in a robust insulin secretory response to hyperglycemia (Heimberg et al., 1995, 1996; Díaz et al., 2007). In the α cell, glucokinase activity (Heimberg et al., 1996) and glycolytic flux (Heimberg et al., 1995) are comparable with that of β cells. However, glucose oxidation (Schuit et al., 1997; Detimary et al., 1998), as well as glucose-induced ATP (Detimary et al., 1998; Ishihara et al., 2003; Ravier and Rutter, 2005; Li et al., 2015), $FADH_2$ (Quesada et al., 2006), and NAD(P)H (Quoix et al., 2009) production are all lower in α cells. Despite α cells having a lower capacity to produce ATP in response to glucose, α cells are still able to retain secretory function at low or even in the complete absence of glucose (De Marinis et al., 2010; Olsen et al., 2005; Salehi et al., 2007; Vieira et al., 2007).

Some tissues have an obligatory need for glucose as an energy substrate (Bélanger et al., 2011). However, many tissues in the body act as ''omnivores'' using a variety of carbon substrates as energy sources to sustain sufficient ATP generation. β-oxidation of free fatty acids (FFAs) is a major energy source in the heart (Most et al., 1969; Lopaschuk et al., 2010), muscle (Rasmussen et al., 2002), and liver (Ontko, 1972). In the islet, FFAs are known to regulate glucose-induced insulin secretion independently of β-oxidation via G-protein-coupled receptor GPR40 signaling (Itoh et al., 2003). In the α cell, less is known about the role of FFAs in regulating glucagon secretion. Although short-term exposure to supra-physiological levels of

 © 2018 The Author(s).
This is an open access article under the CC BY license (http://creativecommons.org/licenses/by/4.0/).

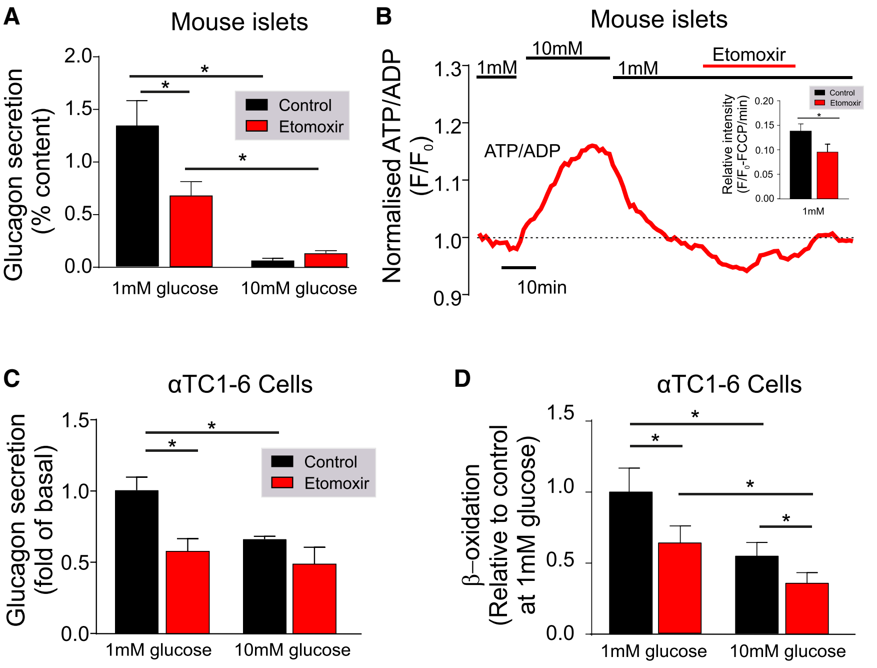

Figure 1. Blocking FFA Transport Pharmacologically or by CPT1a Knockdown Reduces Glucagon Secretion in Mouse α Cells and αTC1-6 Cells

(A) Glucagon secretion from WT mouse islets at 1 and 10 mM glucose with or without etomoxir (100 μM) reduced glucagon secretion (n = 3 mice).

(B) ATP production in WT mouse islets during exposure to 1 or 10 mM glucose, as well as 1 mM glucose and etomoxir (100 μM) (n = 4 mice).

(C) Glucagon secretion from αTC1-6 cells at 1 and 10 mM glucose with or without etomoxir (100 μM) (n = 3).

(D) β-Oxidation measured using [^{3}H]palmitate in αTC1-6 cells at 1 and 10 mM glucose with or without etomoxir (100 μM) (n = 6 observations, from 2 experiments).

All data are represented as mean ± SEM. Paired t test with Tukey post hoc or two-way ANOVA with Student-Newman-Keuls post hoc; *$p < 0.05$. See also Figure S1.

FFA have been demonstrated to increase glucagon secretion (Olofsson et al., 2004), this may be due to GPR40 signaling (Kristinsson et al., 2017), rather than β-oxidation. Circulating FFAs are essential for maintaining systemic energy homeostasis during hypoglycemia (Hue and Taegtmeyer, 2009). Given that β-oxidation of FFAs can provide substantial amounts of ATP, it may be that α cells utilize this energy source, oxidizing FFAs to maintain secretory function in conditions of hypoglycemia.

Here, we show that the oxidation of long-chain FFAs in α cells contributes to maintaining glucagon secretion under hypoglycemic conditions. We also provide a mechanism by which fatty acid oxidation (FAO) energizes glucagon secretion in counter-regulatory conditions.

RESULTS

Glucagon Secretion from Mouse α Cells Depends on FAO

We first explored the dependence of glucagon secretion under hypoglycemic conditions on FAO (Figure 1). Carnitine palmitoyltransferase 1 (CPT1) is a mitochondrial transmembrane enzyme responsible for the formation of acyl-carnitine from long-chain acyl-coenzyme A's (CoA), which can subsequently be transported into the mitochondria and used for β-oxidation. This enzyme is considered rate limiting for β-oxidation of long-chain fatty acids (LCFAs; Kim et al., 2000; Stephens et al., 2007). When mouse islets were exposed to low (1 mM) glucose, pharmacological blockade of CPT1 with etomoxir (100 μM) reduced glucagon secretion by ~40% ($p < 0.001$; Figure 1A) and decreased the cytoplasmic ATP/ADP ratio ($p < 0.0001$; Figure 1B). This was via a direct effect on α cells, because insulin signaling was not altered (p = 0.84; Figure S1). Etomoxir also reduced glucagon secretion by ~40% in αTC1-6 cells at 1 mM glucose (p = 0.005; Figure 1C). This was due to a ~40% reduction in β-oxidation (p = 0.017; Figure 1D). These data show that FAO contributes to glucagon secretion in hypoglycemic conditions.

CPT1a is Essential for Fat Oxidation and Glucagon Secretion in α Cells

We investigated the expression of CPT1 in α cells and found a high degree of co-localization between glucagon and the liver CPT1 isoform (CPT1a) in wild-type (WT) mouse islets (Figure 2A). The muscle CPT1 isoform (CPT1b) is also known to be expressed in α cells, but to a lower degree than CPT1a (Benner et al., 2014). Knockdown of *Cpt1a* in αTC1-6 cells (40% reduced protein content; Figure 2B) resulted in a decrease in glucagon secretion (p = 0.003; Figure 2C) and β-oxidation (p = 0.033; Figure 2D) at 1 mM glucose, consistent with our pharmacological blockade of CPT1 with etomoxir (Figure 1).

The αTC1-6 cell line is an artificial system, and so to understand the implications of reduced CPT1a expression in whole islets, we generated an α cell-specific *Cpt1a* knockout (αCPT1a-KO) mouse. Islets from these mice have no CPT1a expression in 85% ± 2% of the α cells (Figures 2E and 2F). Knockout of *Cpt1a* reduced glucagon secretion by ~40% at 1 mM glucose ($p < 0.001$; Figure 2H), without affecting glucagon content (p = 0.937; Figure 2G) or glucagon secretion at 10 mM glucose (p = 0.64; Figure 2H). Insulin secretion was not affected by the loss of *Cpt1a* in α cells (p = 0.43; Figure S1B). Blood glucose is unlikely to reach 1 mM *in vivo*; therefore, we investigated glucagon secretion *in situ* from control and αCPT1a-KO by perfusion of the whole pancreas with 4, 6, and 10 mM glucose. Glucagon secretion from αCPT1a-KO was significantly lower compared with control mice (p = 0.003; Figures 2I and 2J), and only control mice responded by significantly lowering glucagon secretion in response to 10 mM glucose (p = 0.045; Figures 2I and 2J). These data suggest that *Cpt1a* knockout

OPEN ACCESS
CellPress

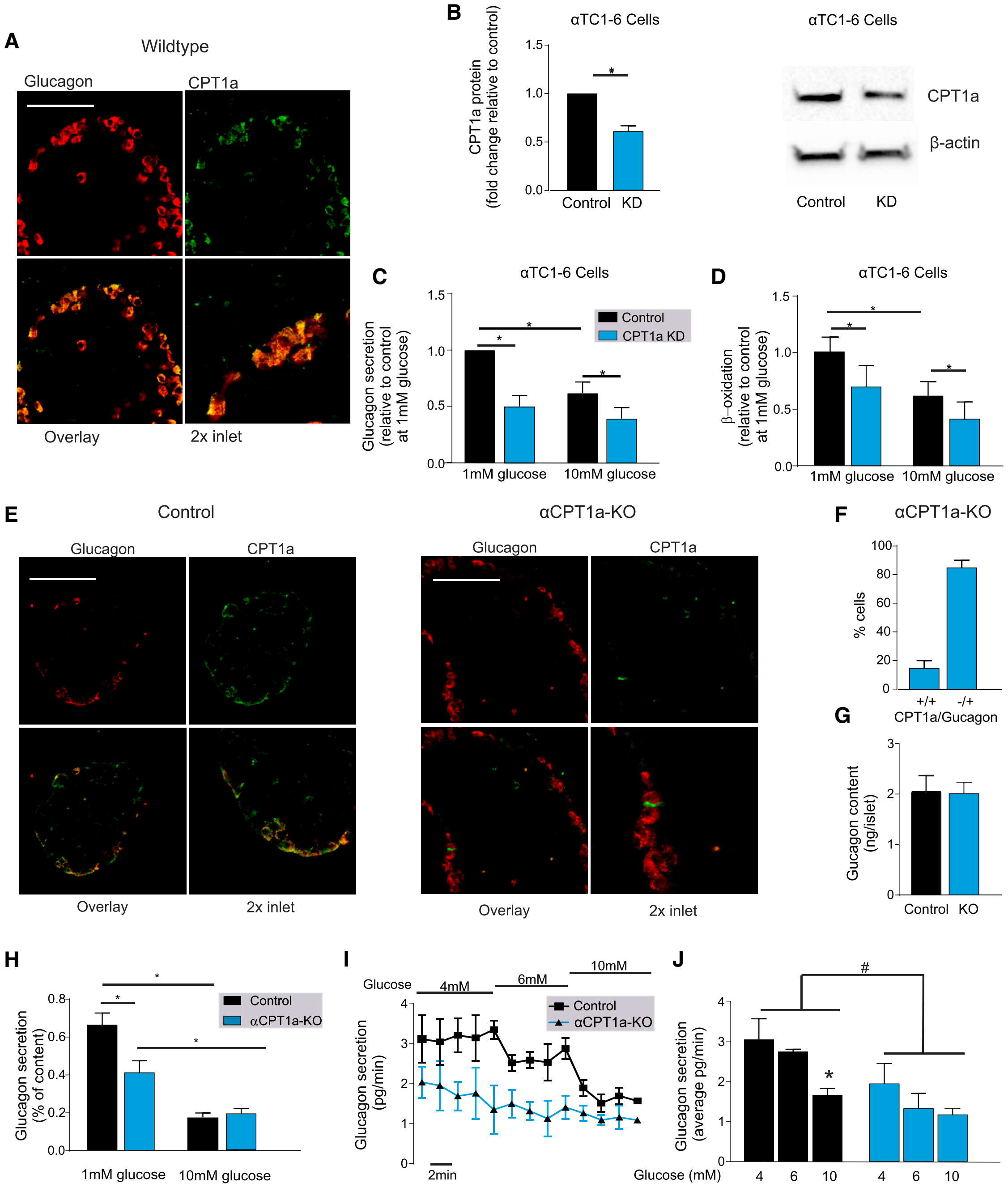

Figure 2. Knockout of CPT1a Specifically in α Cells Reduces Glucagon Secretion

(A) Immunofluorescence for glucagon (red) and CPT1a (green) in WT mouse islets from pancreatic slides. Scale bar, 50 μM.

(B) Knockdown (KD) of CPT1a in αTC1-6 cells treated with either scrambled small interfering RNA (siRNA; control) or siRNA targeting *Cpt1a* mRNA (note the ~40% reduction) (n = 6 observations from 3 experiments).

(C) Glucagon secretion in αTC1-6 cells at 1 and 10 mM glucose treated with either scrambled siRNA (control) or siRNA targeting *Cpt1a* mRNA (n = 3).

(D) β-Oxidation measured using [^{3}H]palmitate in αTC1-6 cells at 1 and 10 mM glucose treated with either scrambled siRNA (control) or siRNA targeting *Cpt1a* mRNA (n = 6 observations, from 2 experiments).

(E) Immunofluorescent detection of glucagon (red) and CPT1a (green) in isolated islets from α cell-specific knockout of *Cpt1a* (αCPT1a-KO) and littermate control mice. Scale bar, 50 μM (n = 5).

(F) Percentage (%) of cells that show co-localization of CPT1a and glucagon in αCPT1a-KO mice (n = 5 islets, 3 mice, 145 cells).

(G) Glucagon content in isolated islets from control and αCPT1a-KO (KO) islets (n = 6).

(H) Glucagon secretion from isolated islets from control and αCPT1a-KO mice at 1 and 10 mM glucose (n = 6).

(legend continued on next page)

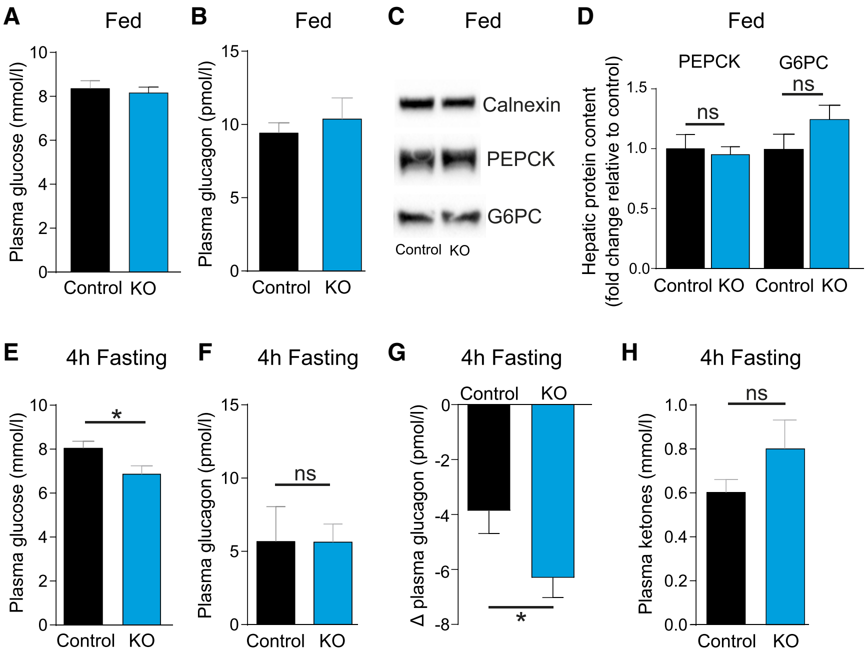

Figure 3. *Cpt1a* Knockout Reduces Fasting Plasma Glucose

(A) Plasma glucose in fed control and αCPT1a-KO (KO) mice (n = 14).

(B) Plasma glucagon in fed control and KO mice (n = 4–5).

(C) Representative blots of PEPCK, G6PC, and calnexin in fed control and KO mice.

(D) Protein content of hepatic PEPCK and G6PC in fed control and KO mice (n = 3–5).

(E) Plasma glucose in control and KO mice following a 4-hr fast (n = 10–12).

(F) Plasma glucagon in control and KO mice following a 4-hr fast (n = 4–5).

(G) Difference between 4 hr fasted and fed plasma glucagon in control and KO mice (n = 4–5).

(H) Plasma ketone bodies in control and KO mice following a 4-hr fast (n = 7–9).

All data are represented as mean ± SEM. Paired t test with Tukey post hoc; *$p < 0.05$. ns, not significant. See also Figure S1.

reduces glucagon secretion from mouse islets in conditions of hypoglycemia.

Cpt1a Knockout Reduces Fasting Plasma Glucose

To test whether FAO contributes to the counter-regulatory response to hypoglycemia *in vivo*, we measured plasma glucose and glucagon in αCPT1a-KO mice (Figure 3). Knockout of *Cpt1a* did not change fed plasma glucose (p = 0.669; Figure 3A), glucagon (p = 0.608; Figure 3B), or hepatic protein content of the gluconeogenic enzymes phosphoenolpyruvate carboxykinase (PEPCK; p = 0.999) and glucose 6-phosphatase (G6PC; p = 0.25; Figures 3C and 3D). Following a 4-hr fast, plasma glucose was decreased in αCPT1a-KO mice compared with control mice (p = 0.03; Figure 3E). Despite plasma glucose being lower in αCPT1a-KO compared with control mice following a 4-hr fast, plasma glucagon was no different between the two genotypes (p = 0.987; Figure 3F). In line with previous studies (Allister et al., 2013), 4 hr of fasting seemed to reduced plasma glucagon in the fed state in both αCPT1a-KO and control mice. The change in plasma glucagon relative to the fed state was greater in αCPT1a-KO compared with control mice (p = 0.029; Figure 3G), indicating a change in the regulation of circulating glucagon levels. We next tested whether ketone body metabolism could contribute to the maintenance of glucagon secretion in WT islets during hypoglycemia. Treating WT islets with 3-hydroxybuterate (0.5 mM) reduced glucagon secretion at 1 mM glucose (p = 0.0002; Figure S1J). This is in agreement with studies from perfused rat pancreas and human subjects (Ikeda et al., 1987; Quabbe et al., 1983) where glucagon secretion was decreased or unaffected by ketones bodies. It is therefore unlikely that ketone metabolism in α cells of the αCPT1a-KO mice could have compensated for the reduced FAO. In support of this, the concentration of fasted plasma ketone bodies was not different in αCPT1a-KO and control mice (p = 0.21; Figure 3H). These data demonstrate that FAO in α cells may contribute to maintaining blood glucose during a 4-hr fast, and that this response is impaired in αCPT1a-KO mice.

Disruption of FAO Changes Electrical Activity in α Cells

α cells are electrically active, utilizing these electrical signals to drive glucagon secretion (Barg et al., 2000; Gromada et al., 2007; MacDonald et al., 2007; Jacobson et al., 2009; Ramracheya et al., 2010; Zhang et al., 2013; Gylfe and Gilon, 2014). Given that inhibition of FAO decreased glucagon secretion (Figure 2), we hypothesized that this was due to an effect on α cell membrane potential.

Inhibition of FAO resulted in a reduction in action potential (AP) amplitude in mouse α cells (Figure 4). Etomoxir (100 μM) reduced AP amplitude ($p < 0.0001$; Figures 4A–4D) and depolarized the minimum membrane potential by 7 mV ($p < 0.001$; Figure 4E). Etomoxir also reduced the amplitude of intracellular Ca^{2+} oscillations (Figure S2). The effect of pharmacological blockade of FAO on membrane potential in α cells was mirrored in αCPT1a-KO mice (Figures 4F–4M). α cells from αCPT1a-KO mice had a lower AP amplitude compared with control mice ($p < 0.0001$; Figure 4H). Minimal membrane potential in αCPT1a-KO mice was depolarized compared with controls (p = 0.026; Figure 4I). The frequency of AP firing was not changed (p = 0.94; Figure 4J), suggesting that disruption of

(I) Glucagon secretion during perfusion in control (n = 3) and αCPT1a-KO (n = 3) mice at 4, 6, and 10 mM glucose.

(J) Average glucagon secretion from control (n = 3) and αCPT1a-KO mice (n = 3) at 4, 6, and 10 mM glucose, calculated from the last 8–10 min in each condition. Two-way ANOVA with post hoc: #$p < 0.05$ versus control; *$p < 0.05$ versus 4 mM glucose.

Paired t test with Tukey post hoc or two-way ANOVA; *$p < 0.05$. See also Figure S1.

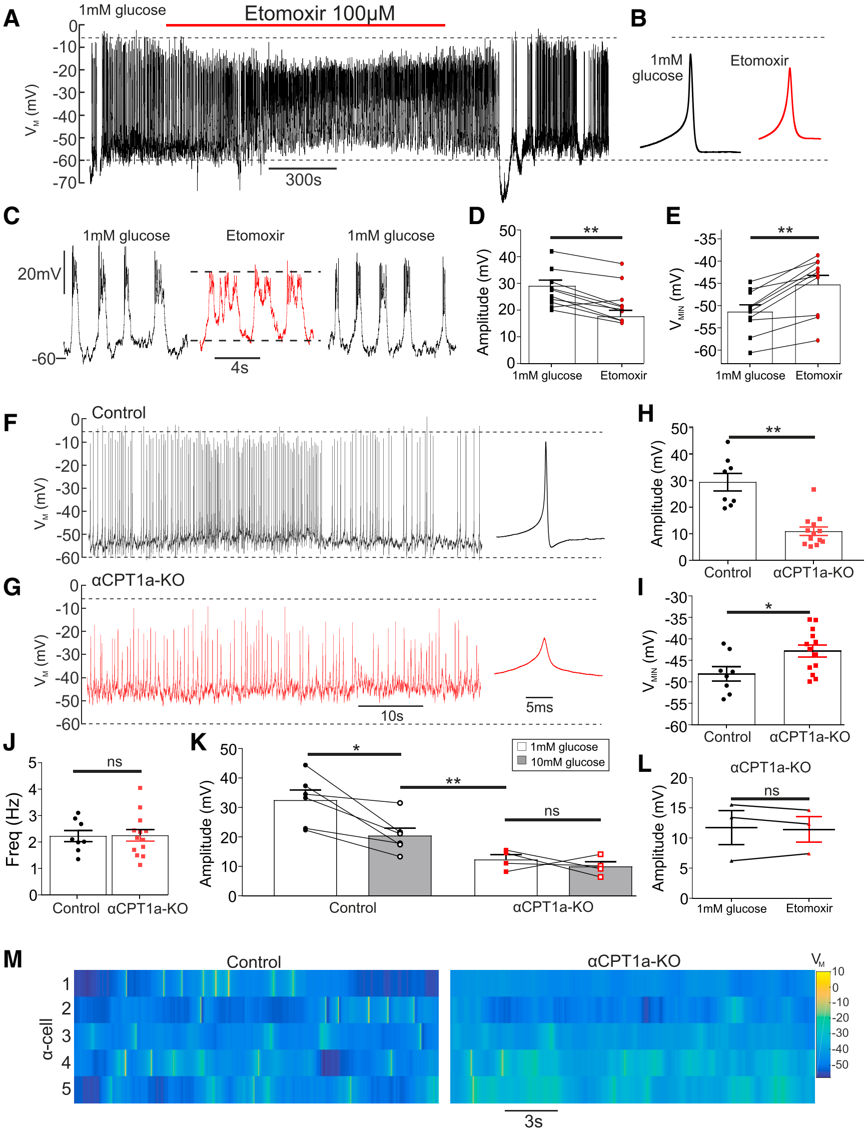

Figure 4. Disruption of FFA Transport by CPT1a Reduces Action Potential Amplitude in Mouse α Cells

(A) Electrical activity in WT α cell at 1 mM glucose with or without etomoxir (100 μM) (10 islets from 6 mice).

(B) Average action potential waveform for (A), in etomoxir compared with 1 mM glucose, measured over the entire experimental condition.

(C) Expanded view on 1 mM glucose and etomoxir conditions for (A).

(D) Action potential amplitude in WT α cells at 1 mM glucose with or without etomoxir (100 μM) (10 islets from 6 WT mice).

(E) Minimum membrane potential (V_{MIN}) in WT α cells at 1 mM glucose with or without etomoxir (100 μM) (10 islets from 6 mice).

(F) Electrical activity in control α cell at 1 mM glucose.

(G) Electrical activity in αCPT1a-KO α cell at 1 mM glucose.

(H) Action potential amplitude in α cells from αCPT1a-KO compared with control at 1 mM glucose (8 control islets for 4 mice and 13 αCPT1a-KO islets for 4 mice).

(I) Minimal potential (V_{MIN}) in α cells from αCPT1a-KO mice compared with control at 1 mM glucose (8 control islets for 4 mice and 13 αCPT1a-KO islets for 4 mice).

(J) Firing frequency in control and αCPT1a-KO islets at 1 mM glucose (8 control islets for 4 mice) and 13 αCPT1a-KO islets for 4 mice).

(K) Action potential amplitude in α cells from control and αCPT1a-KO islets at 1 and 10 mM glucose (6 control islets for 4 mice and 6 αCPT1a-KO islets for 4 mice).

(L) Action potential amplitude in α cells from αCPT1a-KO islets at 1 mM glucose with or without etomoxir (100 μM) (3 islets from 3 mice for each genotype).

(M) Raster plots demonstrating robust action potentials in 5 control α cells, and weaker action potentials in 5 α cells from αCPT1a-KO islets.

Paired t test with Tukey post hoc; *p < 0.05; **p < 0.01; ***p < 0.001. See also Figures S1 and S2.

FAO reduces glucagon secretion by reducing AP amplitude, rather than firing frequency. High glucose (>6 mM) is known to decrease glucagon secretion by reducing AP amplitude in α cells by ~17 mV (Zhang et al., 2013). Consistent with this, in control mice, high glucose (10 mM) decreased AP amplitude by 12 ± 2 mV compared with 1 mM glucose (p = 0.014; Figure 4K). In contrast, 10 mM glucose did not decrease AP amplitude in αCPT1a-KO mice (p = 0.94). Furthermore, the reduction in AP amplitude in αCPT1a-KO at 1 mM glucose was significantly greater than in control islets due to 10 mM glucose (p = 0.005). Therefore, the suppression of AP amplitude by FAO disruption is physiologically significant.

Disruption of FAO Alters α Cell Membrane Potential via a K_{ATP}-Independent Mechanism

We investigated whether the effect of CPT1 inhibition was due to a decrease in K_{ATP} channel conductance (G_{KATP}). First, we noted that our pharmacological data (Figure 4) appeared incompatible with a K_{ATP}-dependent mechanism because etomoxir decreased intracellular ATP (*[ATP]$_i$*) (Figure 1B), which would result in an increase in G_{KATP}, consequently hyperpolarizing the membrane. Nevertheless, because the K_{ATP} channel is crucial to glucagon secretion (MacDonald et al., 2007; Zhang et al., 2013), we wanted to investigate the dependence (if any) of the effect on K_{ATP} channel activity.

We measured G_{KATP} in response to inhibition of CPT1a (Figure 5). Unexpectedly, inhibition of CPT1a with etomoxir (Figure 5A; p = 0.73; 100 μM) or by gene knockout (Figure 5C; p = 0.85) did not change G_{KATP} in α cells. Because β cells have a larger G_{KATP} (Briant et al., 2017), we used them to further interrogate any pharmacological effects of etomoxir on G_{KATP} (Figures 5E–5H). Etomoxir did not change G_{KATP} in β cells at low glucose (p = 0.96). We mimicked the effects of etomoxir on *[ATP]$_i$* by artificially decreasing *[ATP]$_i$*. To this

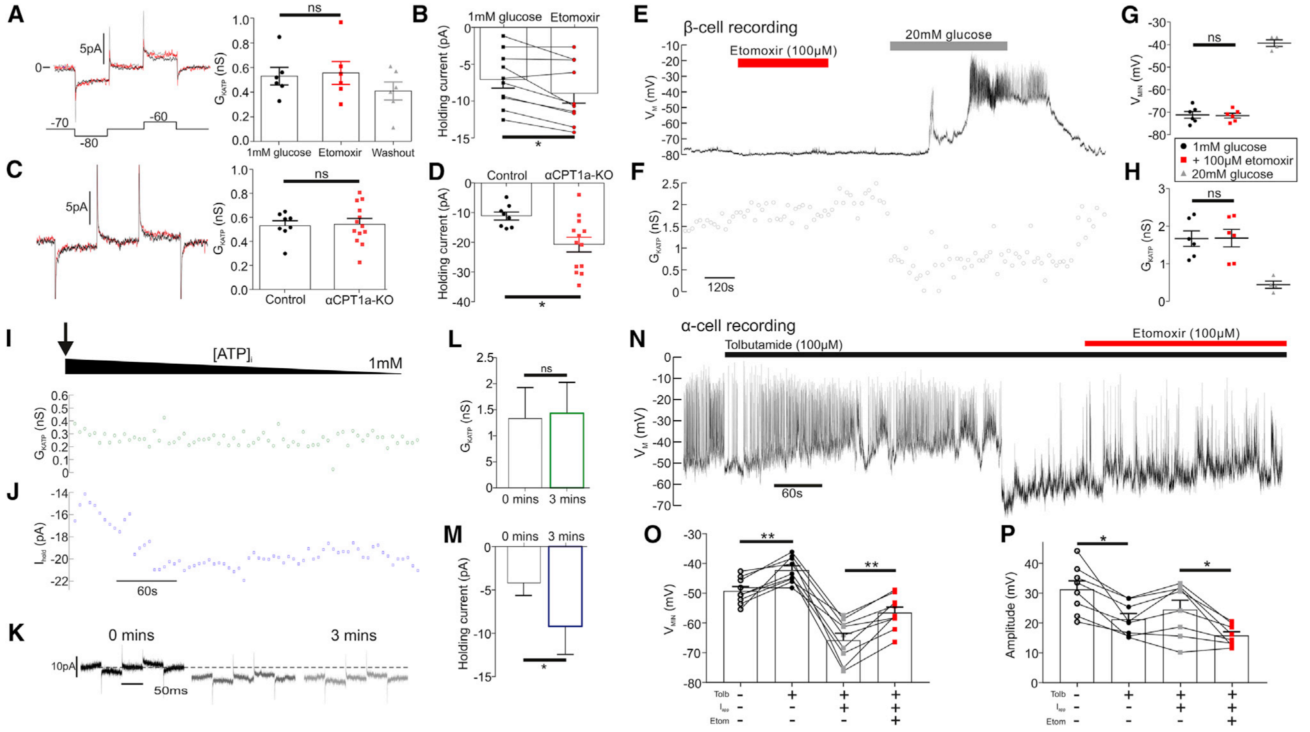

Figure 5. Inhibition of CPT1a Disrupts α Cell Membrane Potential via a K_{ATP}-Independent Mechanism

(A) G_{KATP} in wild-type (WT) mouse α cells at 1 mM glucose with or without etomoxir (100 μM) (6 islets, 5 mice).

(B) Holding current in WT mouse α cells at 1 mM glucose with or without etomoxir (100 μM) (11 islets, 6 mice).

(C) G_{KATP} in α cells from αCPT1a-KO mice compared with control at 1 mM glucose (8 control islets from 4 mice and 13 αCPT1a-KO islets from 4 mice).

(D) Holding current in α cells from αCPT1a-KO mice compared with control at 1 mM glucose (8 control islets for 4 mice and 13 αCPT1a-KO islets for 4 mice).

(E) Electrical activity in WT β cell at 1 mM glucose with or without etomoxir (100 μM) and at 20 mM glucose.

(F) G_{KATP} in WT β cell at 1 mM glucose with or without etomoxir (100 μM) and at 20 mM glucose.

(G) Grouped data of V_M recording in β cells following etomoxir and 20 mM glucose application (6 islets from 6 WT mice).

(H) Grouped data of G_{KATP} recording in β cells following etomoxir and 20 mM glucose application (6 islets from 6 WT mice).

(I) To mimic the effects of etomoxir in 1 mM glucose on *[ATP]i*, we artificially reduced *[ATP]i* by setting the pipette concentration (1 mM) to be lower than the putative *[ATP]i* in 1 mM glucose (>1 mM; Detimary et al., 1998) and measured G_{KATP}. The arrow denotes when whole cell was achieved, which initiates the run-down of *[ATP]i* from the endogenous concentration to the pipette concentration.

(J) Holding current (I_{hold}) from (I).

(K) G_{KATP} and I_{hold} after 0, 1, and 3 min of whole-cell recording (I).

(L) Grouped data, recording G_{KATP} during artificial run-down of *[ATP]i* (4 WT islets, 4 mice).

(M) Grouped data, recording I_{hold} during artificial run-down of *[ATP]i* (4 WT islets, 4 mice).

(N) Membrane potential recording from a WT α cell. Tolbutamide was applied to maximally open K_{ATP} channels. A negative current was then injected to hyperpolarize the α cell. Etomoxir was still able to depolarize α cells.

(O) Grouped data for change in minimum potential (V_{MIN}) (n = 8 islets, n = 4 mice).

(P) Grouped data for change in action potential amplitude (n = 8 islets, n = 4 mice).

Paired t test with Tukey post hoc or two-way ANOVA; *p < 0.05; **p < 0.01; ***p < 0.001. See also Figures S1 and S2.

end, we continuously monitored G_{KATP} in standard whole cell while diluting the cell interior with a pipette solution containing 1 mM ATP (Figures 5I–5M). This would achieve a bona fide decrease in *[ATP]i* because the ATP concentration in α cells at 1 mM glucose is >1 mM (Detimary et al., 1998). Decreasing *[ATP]i* in this way did not change G_{KATP} in α cells (p = 0.93; Figures 5I and 5L), consistent with what has been shown in β cells (Tarasov et al., 2006). In a final set of experiments, we applied etomoxir in the presence of the K_{ATP} channel inhibitor tolbutamide (100 μM; Figure 5N). In these experiments, when the α cells were repolarized by injection of negative currents, etomoxir was still able to depolarize the membrane potential (p = 0.001; Figure 5O) and reduce AP amplitude (p = 0.042; Figure 5P). This effect of etomoxir must be independent of K_{ATP} channel closure. In conclusion, blockade of FAO via inhibition of CPT1 depolarizes the α cell membrane potential by a K_{ATP} channel-independent mechanism.

We therefore sought an ATP-dependent mechanism that could explain these data: namely, result in a depolarization of membrane potential, following a decrease in *[ATP]i*. It can be seen that there was an increase in the holding current (the current required to hold the membrane potential at −70 mV)

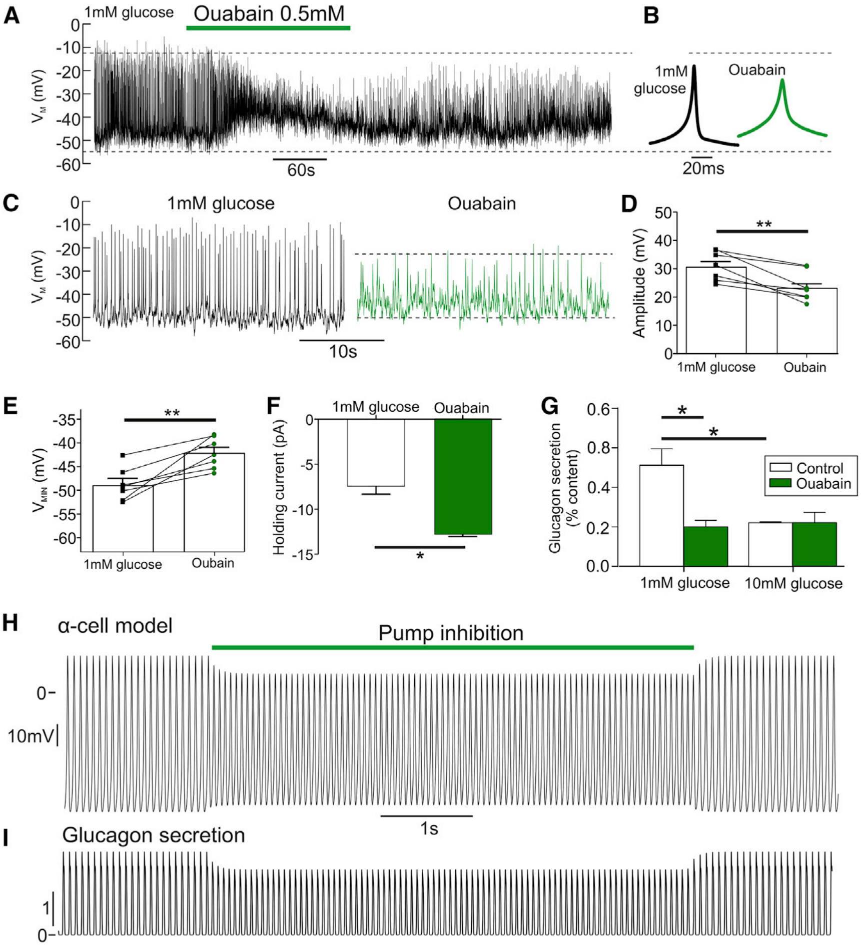

Figure 6. FFA Oxidation Maintains α Cell Membrane Potential by Energizing the Na^+-K^+ Pump

(A) Electrical activity in a WT α cell in response to 1 mM glucose with or without ouabain (0.5 mM).

(B) Average action potential waveform for (A) during 1 mM glucose and ouabain, measured over entire experimental condition.

(C) Expanded view on 1 mM glucose and ouabain conditions for (A).

(D) Grouped data for change in action potential amplitude in response to 1 mM glucose with or without ouabain (0.5 mM; 7 islets, 4 WT mice).

(E) Grouped data for change in minimum membrane potential (V_{MIN}) in WT α cells in response to 1 mM glucose with or without ouabain (0.5 mM; 7 islets, 4 WT mice).

(F) The holding current in absolute value in WT α cells, in response to 1 mM glucose with or without ouabain (0.5 mM; 7 islets, 4 WT mice).

(G) Glucagon secretion from WT islets at 1 and 10 mM glucose with or without ouabain (0.5 mM; n = 4 mice).

(H) Mathematical model of α cell electrical activity demonstrates that a reduction of Na^+-K^+ pump activity (I_{pump}) reduces action potential amplitude, mimicking blockade of CPT1a.

(I) Accompanying model of glucagon secretion demonstrates that this results in a reduction of glucagon secretion, also mimicking blockade of CPT1a.

All data are represented as mean ± SEM. Paired t test with Tukey post hoc or two-way ANOVA with Student-Newman-Keuls post hoc; $^*p < 0.05$. See also Figure S1.

following pharmacological inhibition of CTP1 (p = 0.008; Figure 5B), knockout of *Cpt1a* (p = 0.017; Figure 5D), or by artificially reducing $[ATP]_i$ in 1 mM glucose (Figures 5J–5M; p = 0.049).

FFA Oxidation Maintains α Cell Membrane Potential and Glucagon Secretion by Energizing the Na^+-K^+ Pump

The Na^+-K^+ pump is an ATPase, extruding intracellular Na^+ in exchange for K^+. Its operation is electrogenic and generates an outward current. Thus, reduced activity of the Na^+-K^+ pump would account for the increase in holding current following inhibition of CPT1a (Figures 5B and 5D). We therefore postulated that membrane potential and AP amplitude are maintained in low (1 mM) glucose because FAO energizes the Na^+-K^+ pump.

To investigate this, we exposed islets from WT mice to 0.5 mM ouabain in 1 mM glucose (Figure 6). Blockade of Na^+-K^+ pump activity decreased AP amplitude (p = 0.0005; Figures 6A–6D). Ouabain also reduced glucagon secretion in low glucose (p = 0.0015; Figure 6G), but did not change G_{KATP} in α cells (Figures S1D and S1E) or β cells (p = 0.45; Figures S1F and S1G). Thus, this depolarization was not due to a reduction in G_{KATP}. Ouabain did, however, increase the magnitude of the holding current in both α cells (p = 0.0132; Figure 6F) and β cells (p = 0.0242; Figure S1H). Therefore, all of the effects of FAO inhibition on electrical activity (Figures 4 and 5) were mirrored by blockade of the Na^+-K^+ pump with ouabain. Together, these data suggest that Na^+-K^+ pump activity is high in low glucose, and preservation of this activity by FAO maintains a hyperpolarized membrane potential, and therefore AP amplitude and glucagon secretion, in α cells. To test the consistency of our experimental observations with this theory, we constructed a mathematical model of membrane potential in α cells (Figures 6H and 6I). Reducing energy supply to the Na^+-K^+ pump from FAO in the model recapitulated the experimental data, supporting our hypothesis.

Reduced FAO in Human Islets Causes a Reduction in Electrical Activity and Glucagon Secretion

Islets from human donors stained positive for CPT1a, with co-localization (~90%) of glucagon and CPT1a (Figure 7A). Inhibition of FAO with etomoxir (100 μM) suppressed glucagon secretion at 1 mM glucose (p = 0.008; Figure 7B). Finally, analysis of membrane potential in α cells revealed that etomoxir reduces AP amplitude (p = 0.034; Figures 7C–7E) and depolarizes the membrane (p = 0.035; Figure 7F). These observations recapitulate our findings in mouse islets and suggest that FAO in human α cells contributes to the maintenance of membrane potential and glucagon secretion in low glucose conditions.

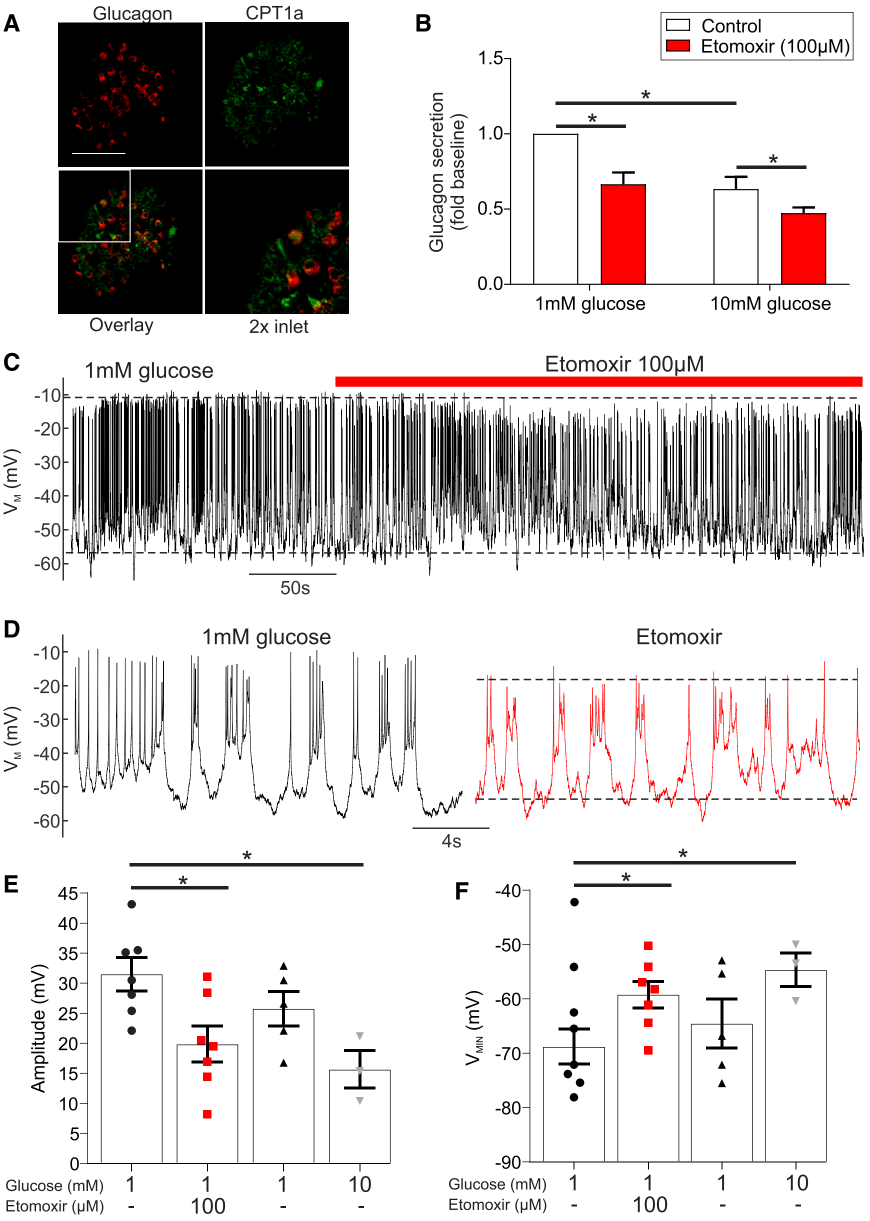

Figure 7. CPT1a Blockade in Human Islets Reduces α Cell Membrane Potential and Glucagon Secretion

(A) Immunofluorescent detection of glucagon (red) and CPT1a (green) in isolated human islets (scale bar, 50 μm).

(B) Glucagon secretion from human islets at 1 and 10 mM glucose with or without etomoxir (100 μM) reduced glucagon secretion (n = 3 donors).

(C) Electrical activity in human α cells at 1 mM glucose with or without etomoxir (100 μM) (n = 3 donors, ≤7 cells).

(D) Expanded view of 1 mM glucose and etomoxir conditions for (C).

(E) Action potential amplitude in human α cells at 1 mM glucose with or without etomoxir (100 μM) and at 10 mM glucose (n = 3 donors, ≤7 cells).

(F) Minimum potential in human α cells at 1 mM glucose with or without etomoxir (100 μM) and at 10 mM glucose (n = 3 donors, ≤7 cells).

All data are represented as mean ± SEM. Paired t test with Tukey post hoc or two-way ANOVA with Student-Newman-Keuls post hoc; *$p < 0.05$.

DISCUSSION

In this study we investigated the role of FAO in regulating glucagon secretion under hypoglycemic conditions. We found that pharmacological blockade or knockout of CPT1a profoundly reduced glucagon secretion from mouse islets at low glucose. These findings were mirrored in human islets with pharmacological inhibition of CPT1. In both mouse and human α cells, inhibition of CPT1 was associated with membrane potential depolarization and reduced AP amplitude.

Our findings suggest that during fasting, when blood glucose becomes low, fatty acids play a significant role in maintaining blood glucose by sustaining basal glucagon secretion. We show that FAO contributes to ATP production in low glucose conditions in α cells, and that this FFA-derived ATP maintains glucagon secretion by energizing the Na^+-K^+ pump. We propose that in low glucose concentrations, ATP generated by FAO is an essential energy supply for the Na^+-K^+ pump, keeping the α cell membrane sufficiently repolarized to prevent voltage-dependent inactivation of the ion channels involved in AP firing, thus allowing the generation of large-amplitude APs. When glucose becomes available, ATP derived from glucose oxidation triggers membrane depolarization by closure of K_{ATP} channels, leading to a K_{ATP}-dependent reduction in glucagon secretion.

The K_{ATP} channel has been demonstrated to mediate the intrinsic regulation of glucagon secretion from α cells (MacDonald et al., 2007; Zhang et al., 2013). According to this model of counter-regulation, a reduction in glucose concentrations causes opening of K_{ATP} channels, a hyperpolarization of membrane potential, an increase in AP amplitude, and, therefore, glucagon secretion. However, this model is not wholly accepted (see Gylfe, 2013, 2016; Gylfe and Gilon, 2014). In low glucose, we observed that inhibition or reduction of FAO depolarized α cells and decreased glucagon secretion. If this depolarizing effect of inhibiting FAO were mediated by changes in K_{ATP} channel activity, we would expect to observe a reduction in K_{ATP} conductance. However, this is incompatible with our data because inhibition of FAO decreases $[ATP]_i$, which would cause an increase in K_{ATP} conductance. Furthermore, we did not observe a change in K_{ATP} conductance in response to CPT1a inhibition, either

pharmacologically or via knockout, suggesting that K_{ATP} channels are not directly involved.

To identify a possible mechanism underlying the effect on α cell electrical activity, we considered energy-consuming cellular mechanisms that result in membrane depolarization following a reduction in cellular ATP. The Na^{+}-K^{+} pump is a major energy consumer in most types of cells (~10% in muscle (Pirkmajer and Chibalin, 2016), ~40% in neurons (Attwell and Laughlin, 2001), and ~50% in the kidney (Clausen et al., 1991), utilizing more ATP than any other enzyme and consuming 19%–28% of whole-body ATP (Rolfe and Brown, 1997). The α cell appears to be no exception; we show that the application of the Na^{+}-K^{+} pump inhibitor ouabain robustly depolarizes α cells. Considering that the Na^{+}-K^{+} pump has a K_m of ~0.4 mM for ATP (Javorková et al., 2009), α cell pump activity at 1 mM glucose would be drastically decreased when ATP levels are further reduced. Furthermore, this K_m is 30 times higher than the K_m of the K_{ATP} channel to ATP (Tarasov et al., 2006; Javorková et al., 2009). The pump would therefore turn off before the channel would be affected by the reduction in $[ATP]_i$. We suggest that the reduction in ATP, which occurs when FAO is blocked with etomoxir or when *Cpt1a* is knocked out in α cells, leads to a reduction in Na^{+}-K^{+} pump activity in low glucose.

Defects in mitochondrial β-oxidation have serious clinical consequences (Kompare and Rizzo, 2008) and account for a major cause of hypoglycemic seizures. There are at least 12 FAO disorders described, of which 10 are associated with routine fasting hypoglycemia (Grosse et al., 2006). CPT1a deficiency presents in infancy, is characterized by hypoketotic hypoglycemia (Ogier de Baulny et al., 1995; Greenberg et al., 2009), and is treated with frequent feedings to prevent prolonged fasting (Longo et al., 2006).

It has previously been suggested that the liver uses ATP generated from FAO to maintain glucose production (Staehr et al., 2003; Lam et al., 2003). However, recent findings suggest that hepatic FAO is expendable for maintaining 24-hr fasting blood glucose (Lee et al., 2016). Thus, loss of CPT1a activity in the liver of CPT1a-deficient patients may cause the hypoketonemia, but not the hypoglycemia. Our data support this and indicate that the hypoglycemia in these patients may be caused by a loss of FAO in the α cell and, consequently, reduced glucagon secretion.

EXPERIMENTAL PROCEDURES

All animal experiments were conducted in accordance with the UK Animals Scientific Procedures Act (1986) and University of Oxford ethical guidelines, and were approved by the local Ethical Committees. Human pancreatic islets were isolated (with ethical approval and clinical consent) at the Diabetes Research and Wellness Foundation Human Islet Isolation Facility (OCDEM, Oxford, UK) from pancreases of six non-diabetic donors. Donors were on average 61 years old (range 25–76) with a BMI of 24 (range 19.3–30) and HbA1c of 5.5% (range 5.3–5.9). Three of six donors were female.

Animals and Generation of αCPT1a-KO Mice

C57BL/6j mice were used as WT mice in this study. To generate αCPT1a-KO, mice carrying a loxP insert flanking exons 11 and 12 of the *Cpt1a* gene (Schoors et al., 2015) were crossed with mice carrying Cre recombinase under the control of the proglucagon promoter (Parker et al., 2012). Mice homozygous for the loxP allele were used as controls and are referred to as such.

In Vivo Measurements of Plasma Glucose, Glucagon, and Ketone Body Concentration

Plasma glucose, glucagon, and ketone body measurements were conducted *in vivo* on αCPT1a-KO and control mice in response to fasting. Mice were restrained, and a tail-vein sample of plasma was used to measure fed plasma glucose and ketone bodies. Mice were then individually caged for the 4-hr fasting period and given unrestricted access to water during this time. At the end of the fasting period, mice were restrained and a tail-vein sample of plasma was used to measure plasma glucose and ketone bodies. Mice were then culled by cervical dislocation and trunk plasma collected. The serum was then removed and stored at −80°C. Serum samples were used to measure plasma glucagon. Measurements were conducted using a dual mouse insulin/glucagon assay system (Meso Scale Discovery, MD, USA), according to the manufacturer's protocol.

Isolation of Pancreatic Islets

Mice at 12–16 weeks of age were killed by cervical dislocation (Schedule 1 procedure). Pancreatic islets were isolated by Liberase digestion followed by manual picking. Islets were used acutely and were, pending the experiments, maintained in tissue culture for <24 hr in RPMI 1640 (11879-020; GIBCO, Thermo Fisher Scientific) containing 1% penicillin/streptomycin (1214-122; GIBCO, Thermo Fisher Scientific), 10% fetal bovine serum (FBS; F7524-500G; Sigma-Aldrich), and 7.5 mM glucose before the measurements.

Hormone Release Measurements

Measurements of insulin and glucagon secretion were performed using sequential incubations of isolated mouse and human islets as described in the Supplemental Information.

Electrophysiology

All electrophysiological measurements were performed at 33°C to 34°C on α cells within intact islets (from αCPT1a-KO mice, littermate controls, WT C57BL/6j mice, and human islets). Membrane potential and whole-cell K_{ATP}-current recordings were conducted using the perforated patch technique, as previously described (De Marinis et al., 2010). The composition of solutions is described in the Supplemental Information.

ATP Imaging

The ATP/ADP sensor Perceval was used, as previously described (Adam et al., 2017).

FFA Oxidation Measurements

αTC1-6 cells were cultured overnight in RPMI culture medium containing 5 mM glucose. On the day of the experiment, the cells were incubated in 0 mM glucose Krebs Ringer buffer (KRB) with or without etomoxir. The cells were then exposed to 0.3 mM palmitate containing 0.22 MBq [^{3}H]palmitate for 1 hr. The supernatant was then subjected to a Falkes extraction and the aqueous phase assayed for H^{3} content. From this, β-oxidation was calculated and normalized to cell count. Further details are in the Supplemental Information.

Mathematical Model of α Cell Membrane Potential

A mathematical model of Na^{+}-K^{+} pump activity was added to a model of α cell membrane potential (Briant et al., 2018) and simulated in the simulation environment NEURON with a 25-μS time step. The model is further described in the Supplemental Information.

Statistical Tests

All data are reported as mean ± SEM, unless otherwise stated. Statistical significance was defined as $p < 0.05$. All statistical tests were conducted in Prism (GraphPad Software, San Diego, CA, USA). For two groupings, a t test was conducted with the appropriate post hoc test. For more than two groupings, a one-way ANOVA was conducted. If there were two independent variables, a two-way ANOVA was conducted. If the data passed normality criteria (D'Agostino's test of normality and Bartlett's test of equal variances), a parametric test was conducted with the appropriate post hoc

test (Tukey or Student-Neumann-Keuls). If the normality criteria were not met, a Kruskal-Wallis test with Dunn's multiple comparison test was conducted.

SUPPLEMENTAL INFORMATION

Supplemental Information includes Supplemental Experimental Procedures and two figures and can be found with this article online at https://doi.org/10.1016/j.celrep.2018.05.035.

ACKNOWLEDGMENTS

We would like to acknowledge Dr. Anne Clark (OCDEM, Oxford, UK), Dr. Andrei Tarasov (OCDEM), Dr. Kerry McLaughlin (OCDEM), and Coleen Lopez (DPAG, Oxford, UK) for technical advice. We would also like to thank Prof. Emeritus Jens Kristian Knudsen (University of Southern Denmark, Denmark), Dr. Laura Arbor (University of Victoria, BC, Canada), and Cheryl Rockman-Greenberg (CHRIM, Winnipeg, MB, Canada) for valuable scientific input. We thank Dr. Patrick MacDonald and the Alberta Diabetes Institute IsletCore (University of Alberta, AB, Canada) for providing human islets, the isolation of which was supported in part by the Alberta Diabetes Foundation, the Human Organ Procurement and Exchange Program (Edmonton), and the Trillium Gift of Life Network (Toronto). We would also like to thank the generosity of the organ donors and their families. This study was funded by the 2015 Goodger & Schorstein Scholarship, a Wellcome Trust Senior Investigator Award (095531), Wellcome Trust Strategic Award (884655), Wellcome Trust programme grant (089795), European Research Council (322620), Swedish Research Council, and the Knut and Alice Wallenberg's Stiftelse. L.J.B.B. is supported by a Sir Henry Wellcome Postdoctoral Fellowship (Wellcome Trust, 201325/Z/16/Z) and a Junior Research Fellowship from Trinity College, Oxford. J.K.G. and M.S.D. are supported by a Novo Nordisk postdoctoral fellowship run in partnership with the University of Oxford.

AUTHOR CONTRIBUTIONS

Conceptualization, L.J.B.B. and J.G.K.; Software, L.J.B.B.; Methodology, L.J.B.B., J.G.K., and P.R.; Investigation, L.J.B.B., J.G.K., M.S.D., M.V.C., and N.J.G.R.; Writing – Original Draft, L.J.B.B. and J.G.K.; Writing – Review & Editing, L.J.B.B., J.G.K., P.R., P.R.V.J., P.C., M.S.D., M.V.C., and N.J.G.R.; Funding Acquisition, L.J.B.B., J.G.K., and P.R.; Resources, P.R.V.J. and P.C.; Supervision, L.J.B.B. and J.G.K.

DECLARATION OF INTERESTS

The authors declare no competing interests.

Received: January 30, 2018
Revised: March 19, 2018
Accepted: May 11, 2018
Published: June 12, 2018

REFERENCES

Adam, J., Ramracheya, R., Chibalina, M.V., Ternette, N., Hamilton, A., Tarasov, A.I., Zhang, Q., Rebelato, E., Rorsman, N.J.G., Martín-Del-Río, R., et al. (2017). Fumarate hydratase deletion in pancreatic β cells leads to progressive diabetes. Cell Rep. *20*, 3135–3148.

Allister, E.M., Robson-Doucette, C.A., Prentice, K.J., Hardy, A.B., Sultan, S., Gaisano, H.Y., Kong, D., Gilon, P., Herrera, P.L., Lowell, B.B., and Wheeler, M.B. (2013). UCP2 regulates the glucagon response to fasting and starvation. Diabetes *62*, 1623–1633.

Attwell, D., and Laughlin, S.B. (2001). An energy budget for signaling in the grey matter of the brain. J. Cereb. Blood Flow Metab. *21*, 1133–1145.

Barg, S., Galvanovskis, J., Göpel, S.O., Rorsman, P., and Eliasson, L. (2000). Tight coupling between electrical activity and exocytosis in mouse glucagon-secreting alpha-cells. Diabetes *49*, 1500–1510.

Bélanger, M., Allaman, I., and Magistretti, P.J. (2011). Brain energy metabolism: focus on astrocyte-neuron metabolic cooperation. Cell Metab. *14*, 724–738.

Benner, C., van der Meulen, T., Caceres, E., Tigyi, K., Donaldson, C.J., and Huising, M.O. (2014). The transcriptional landscape of mouse beta cells compared to human beta cells reveals notable species differences in long non-coding RNA and protein-coding gene expression. BMC Genomics *15*, 620.

Bolli, G.B., and Perriello, G. (1990). Impact of activated glucose counterregulation on insulin requirements in insulin-dependent diabetes mellitus. Horm. Metab. Res. Suppl. *24*, 87–96.

Briant, L.J., Zhang, Q., Vergari, E., Kellard, J.A., Rodriguez, B., Ashcroft, F.M., and Rorsman, P. (2017). Functional identification of islet cell types by electrophysiological fingerprinting. J. R. Soc. Interface *14*, 20160999.

Briant, L.J.B., Reinbothe, T.M., Spiliotis, I., Miranda, C., Rodriguez, B., and Rorsman, P. (2018). δ-Cells and β-cells are electrically coupled and regulate α-cell activity via somatostatin. J. Physiol. *596*, 197–215.

Caicedo, A. (2013). Paracrine and autocrine interactions in the human islet: more than meets the eye. Semin. Cell Dev. Biol. *24*, 11–21.

Clausen, T., Van Hardeveld, C., and Everts, M.E. (1991). Significance of cation transport in control of energy metabolism and thermogenesis. Physiol. Rev. *71*, 733–774.

Cryer, P.E. (2002). Hypoglycaemia: the limiting factor in the glycaemic management of Type I and Type II diabetes. Diabetologia *45*, 937–948.

Cryer, P.E. (2008). Glucagon and hyperglycaemia in diabetes. Clin. Sci. (Lond.) *114*, 589–590.

Cryer, P.E., Davis, S.N., and Shamoon, H. (2003). Hypoglycemia in diabetes. Diabetes Care *26*, 1902–1912.

D'Alessio, D. (2011). The role of dysregulated glucagon secretion in type 2 diabetes. Diabetes Obes. Metab. *13* (*Suppl 1*), 126–132.

De Marinis, Y.Z., Salehi, A., Ward, C.E., Zhang, Q., Abdulkader, F., Bengtsson, M., Braha, O., Braun, M., Ramracheya, R., Amisten, S., et al. (2010). GLP-1 inhibits and adrenaline stimulates glucagon release by differential modulation of N- and L-type Ca2+ channel-dependent exocytosis. Cell Metab. *11*, 543–553.

Detimary, P., Dejonghe, S., Ling, Z., Pipeleers, D., Schuit, F., and Henquin, J.C. (1998). The changes in adenine nucleotides measured in glucose-stimulated rodent islets occur in beta cells but not in alpha cells and are also observed in human islets. J. Biol. Chem. *273*, 33905–33908.

Díaz, M., Antonescu, C.N., Capilla, E., Klip, A., and Planas, J.V. (2007). Fish glucose transporter (GLUT)-4 differs from rat GLUT4 in its traffic characteristics but can translocate to the cell surface in response to insulin in skeletal muscle cells. Endocrinology *148*, 5248–5257.

Dunning, B.E., Foley, J.E., and Ahrén, B. (2005). Alpha cell function in health and disease: influence of glucagon-like peptide-1. Diabetologia *48*, 1700–1713.

Gerich, J.E., Langlois, M., Noacco, C., Karam, J.H., and Forsham, P.H. (1973). Lack of glucagon response to hypoglycemia in diabetes: evidence for an intrinsic pancreatic alpha cell defect. Science *182*, 171–173.

Greenberg, C.R., Dilling, L.A., Thompson, G.R., Seargeant, L.E., Haworth, J.C., Phillips, S., Chan, A., Vallance, H.D., Waters, P.J., Sinclair, G., et al. (2009). The paradox of the carnitine palmitoyltransferase type Ia P479L variant in Canadian Aboriginal populations. Mol. Genet. Metab. *96*, 201–207.

Gromada, J., Franklin, I., and Wollheim, C.B. (2007). Alpha-cells of the endocrine pancreas: 35 years of research but the enigma remains. Endocr. Rev. *28*, 84–116.

Grosse, S.D., Khoury, M.J., Greene, C.L., Crider, K.S., and Pollitt, R.J. (2006). The epidemiology of medium chain acyl-CoA dehydrogenase deficiency: an update. Genet. Med. *8*, 205–212.

Gylfe, E. (2013). Glucose control of glucagon secretion: there is more to it than KATP channels. Diabetes *62*, 1391–1393.

Gylfe, E. (2016). Glucose control of glucagon secretion—'There's a brand-new gimmick every year'. Ups. J. Med. Sci. *121*, 120–132.

Gylfe, E., and Gilon, P. (2014). Glucose regulation of glucagon secretion. Diabetes Res. Clin. Pract. *103*, 1–10.

Heimberg, H., De Vos, A., Pipeleers, D., Thorens, B., and Schuit, F. (1995). Differences in glucose transporter gene expression between rat pancreatic alpha- and beta-cells are correlated to differences in glucose transport but not in glucose utilization. J. Biol. Chem. *270*, 8971–8975.

Heimberg, H., De Vos, A., Moens, K., Quartier, E., Bouwens, L., Pipeleers, D., Van Schaftingen, E., Madsen, O., and Schuit, F. (1996). The glucose sensor protein glucokinase is expressed in glucagon-producing alpha-cells. Proc. Natl. Acad. Sci. USA *93*, 7036–7041.

Hue, L., and Taegtmeyer, H. (2009). The Randle cycle revisited: a new head for an old hat. Am. J. Physiol. Endocrinol. Metab. *297*, E578–E591.

Ikeda, T., Yoshida, T., Ito, Y., Murakami, I., Mokuda, O., Tominaga, M., and Mashiba, H. (1987). Effect of beta-hydroxybutyrate and acetoacetate on insulin and glucagon secretion from perfused rat pancreas. Arch. Biochem. Biophys. *257*, 140–143.

Ishihara, H., Maechler, P., Gjinovci, A., Herrera, P.L., and Wollheim, C.B. (2003). Islet beta-cell secretion determines glucagon release from neighbouring alpha-cells. Nat. Cell Biol. *5*, 330–335.

Itoh, Y., Kawamata, Y., Harada, M., Kobayashi, M., Fujii, R., Fukusumi, S., Ogi, K., Hosoya, M., Tanaka, Y., Uejima, H., et al. (2003). Free fatty acids regulate insulin secretion from pancreatic beta cells through GPR40. Nature *422*, 173–176.

Jacobson, D.A., Wicksteed, B.L., and Philipson, L.H. (2009). The alpha-cell conundrum: ATP-sensitive K+ channels and glucose sensing. Diabetes *58*, 304–306.

Javorková, V., Mézesová, L., Vlkovicová, J., and Vrbjar, N. (2009). Acute diabetes mellitus and its influence on renal Na,K-ATPase in both genders. Gen. Physiol. Biophys. *28*, 39–46.

Kim, J.Y., Hickner, R.C., Cortright, R.L., Dohm, G.L., and Houmard, J.A. (2000). Lipid oxidation is reduced in obese human skeletal muscle. Am. J. Physiol. Endocrinol. Metab. *279*, E1039–E1044.

Kompare, M., and Rizzo, W.B. (2008). Mitochondrial fatty-acid oxidation disorders. Semin. Pediatr. Neurol. *15*, 140–149.

Kristinsson, H., Sargsyan, E., Manell, H., Smith, D.M., Göpel, S.O., and Bergsten, P. (2017). Basal hypersecretion of glucagon and insulin from palmitate-exposed human islets depends on FFAR1 but not decreased somatostatin secretion. Sci. Rep. *7*, 4657.

Lam, T.K., Carpentier, A., Lewis, G.F., van de Werve, G., Fantus, I.G., and Giacca, A. (2003). Mechanisms of the free fatty acid-induced increase in hepatic glucose production. Am. J. Physiol. Endocrinol. Metab. *284*, E863–E873.

Leahy, J.L. (2005). Pathogenesis of type 2 diabetes mellitus. Arch. Med. Res. *36*, 197–209.

Lee, J., Choi, J., Scafidi, S., and Wolfgang, M.J. (2016). Hepatic fatty acid oxidation restrains systemic catabolism during starvation. Cell Rep. *16*, 201–212.

Li, J., Yu, Q., Ahooghalandari, P., Gribble, F.M., Reimann, F., Tengholm, A., and Gylfe, E. (2015). Submembrane ATP and Ca2+ kinetics in α-cells: unexpected signaling for glucagon secretion. FASEB J. *29*, 3379–3388.

Longo, N., Amat di San Filippo, C., and Pasquali, M. (2006). Disorders of carnitine transport and the carnitine cycle. Am. J. Med. Genet. C. Semin. Med. Genet. *142C*, 77–85.

Lopaschuk, G.D., Ussher, J.R., Folmes, C.D.L., Jaswal, J.S., and Stanley, W.C. (2010). Myocardial fatty acid metabolism in health and disease. Physiol. Rev. *90*, 207–258.

MacDonald, P.E., De Marinis, Y.Z., Ramracheya, R., Salehi, A., Ma, X., Johnson, P.R.V., Cox, R., Eliasson, L., and Rorsman, P. (2007). A K ATP channel-dependent pathway within alpha cells regulates glucagon release from both rodent and human islets of Langerhans. PLoS Biol. *5*, e143.

Most, A.S., Brachfeld, N., Gorlin, R., and Wahren, J. (1969). Free fatty acid metabolism of the human heart at rest. J. Clin. Invest. *48*, 1177–1188.

Ogier de Baulny, H., Slama, A., Touati, G., Turnbull, D.M., Pourfarzam, M., and Brivet, M. (1995). Neonatal hyperammonemia caused by a defect of carnitine-acylcarnitine translocase. J. Pediatr. *127*, 723–728.

Olofsson, C.S., Salehi, A., Göpel, S.O., Holm, C., and Rorsman, P. (2004). Palmitate stimulation of glucagon secretion in mouse pancreatic alpha-cells results from activation of L-type calcium channels and elevation of cytoplasmic calcium. Diabetes *53*, 2836–2843.

Olsen, H.L., Theander, S., Bokvist, K., Buschard, K., Wollheim, C.B., and Gromada, J. (2005). Glucose stimulates glucagon release in single rat alpha-cells by mechanisms that mirror the stimulus-secretion coupling in beta-cells. Endocrinology *146*, 4861–4870.

Ontko, J.A. (1972). Metabolism of free fatty acids in isolated liver cells. Factors affecting the partition between esterification and oxidation. J. Biol. Chem. *247*, 1788–1800.

Parker, H.E., Adriaenssens, A., Rogers, G., Richards, P., Koepsell, H., Reimann, F., and Gribble, F.M. (2012). Predominant role of active versus facilitative glucose transport for glucagon-like peptide-1 secretion. Diabetologia *55*, 2445–2455.

Pirkmajer, S., and Chibalin, A.V. (2016). Na,K-ATPase regulation in skeletal muscle. Am. J. Physiol. Endocrinol. Metab. *311*, E1–E31.

Quabbe, H.J., Trompke, M., and Luyckx, A.S. (1983). Influence of ketone body infusion on plasma growth hormone and glucagon in man. J. Clin. Endocrinol. Metab. *57*, 613–618.

Quesada, I., Todorova, M.G., and Soria, B. (2006). Different metabolic responses in alpha-, beta-, and delta-cells of the islet of Langerhans monitored by redox confocal microscopy. Biophys. J. *90*, 2641–2650.

Quoix, N., Cheng-Xue, R., Mattart, L., Zeinoun, Z., Guiot, Y., Beauvois, M.C., Henquin, J.C., and Gilon, P. (2009). Glucose and pharmacological modulators of ATP-sensitive K+ channels control [Ca2+]c by different mechanisms in isolated mouse alpha-cells. Diabetes *58*, 412–421.

Ramracheya, R., Ward, C., Shigeto, M., Walker, J.N., Amisten, S., Zhang, Q., Johnson, P.R., Rorsman, P., and Braun, M. (2010). Membrane potential-dependent inactivation of voltage-gated ion channels in alpha-cells inhibits glucagon secretion from human islets. Diabetes *59*, 2198–2208.

Rasmussen, B.B., Holmbäck, U.C., Volpi, E., Morio-Liondore, B., Paddon-Jones, D., and Wolfe, R.R. (2002). Malonyl coenzyme A and the regulation of functional carnitine palmitoyltransferase-1 activity and fat oxidation in human skeletal muscle. J. Clin. Invest. *110*, 1687–1693.

Ravier, M.A., and Rutter, G.A. (2005). Glucose or insulin, but not zinc ions, inhibit glucagon secretion from mouse pancreatic alpha-cells. Diabetes *54*, 1789–1797.

Rolfe, D.F., and Brown, G.C. (1997). Cellular energy utilization and molecular origin of standard metabolic rate in mammals. Physiol. Rev. *77*, 731–758.

Salehi, A., Qader, S.S., Grapengiesser, E., and Hellman, B. (2007). Pulses of somatostatin release are slightly delayed compared with insulin and antisynchronous to glucagon. Regul. Pept. *144*, 43–49.

Schoors, S., Bruning, U., Missiaen, R., Queiroz, K.C.S., Borgers, G., Elia, I., Zecchin, A., Cantelmo, A.R., Christen, S., Goveia, J., et al. (2015). Fatty acid carbon is essential for dNTP synthesis in endothelial cells. Nature *520*, 192–197.

Schuit, F., De Vos, A., Farfari, S., Moens, K., Pipeleers, D., Brun, T., and Prentki, M. (1997). Metabolic fate of glucose in purified islet cells. Glucose-regulated anaplerosis in beta cells. J. Biol. Chem. *272*, 18572–18579.

Shamoon, H., Friedman, S., Canton, C., Zacharowicz, L., Hu, M., and Rossetti, L. (1994). Increased epinephrine and skeletal muscle responses to hypoglycemia in non-insulin-dependent diabetes mellitus. J. Clin. Invest. *93*, 2562–2571.

Staehr, P., Hother-Nielsen, O., Landau, B.R., Chandramouli, V., Holst, J.J., and Beck-Nielsen, H. (2003). Effects of free fatty acids per se on glucose production, gluconeogenesis, and glycogenolysis. Diabetes *52*, 260–267.

Stephens, F.B., Constantin-Teodosiu, D., and Greenhaff, P.L. (2007). New insights concerning the role of carnitine in the regulation of fuel metabolism in skeletal muscle. J. Physiol. *581*, 431–444.

Tarasov, A.I., Girard, C.A., and Ashcroft, F.M. (2006). ATP sensitivity of the ATP-sensitive K+ channel in intact and permeabilized pancreatic beta-cells. Diabetes *55*, 2446–2454.

Unger, R.H., and Cherrington, A.D. (2012). Glucagonocentric restructuring of diabetes: a pathophysiologic and therapeutic makeover. J. Clin. Invest. *122*, 4–12.

Vieira, E., Salehi, A., and Gylfe, E. (2007). Glucose inhibits glucagon secretion by a direct effect on mouse pancreatic alpha cells. Diabetologia *50*, 370–379.

Zhang, Q., Ramracheya, R., Lahmann, C., Tarasov, A., Bengtsson, M., Braha, O., Braun, M., Brereton, M., Collins, S., Galvanovskis, J., et al. (2013). Role of KATP channels in glucose-regulated glucagon secretion and impaired counterregulation in type 2 diabetes. Cell Metab. *18*, 871–882.

Cell Reports, Volume *23*

Supplemental Information

CPT1a-Dependent Long-Chain Fatty Acid Oxidation Contributes to Maintaining Glucagon Secretion from Pancreatic Islets

Linford J.B. Briant, Michael S. Dodd, Margarita V. Chibalina, Nils J.G. Rorsman, Paul R.V. Johnson, Peter Carmeliet, Patrik Rorsman, and Jakob G. Knudsen

Figure S1

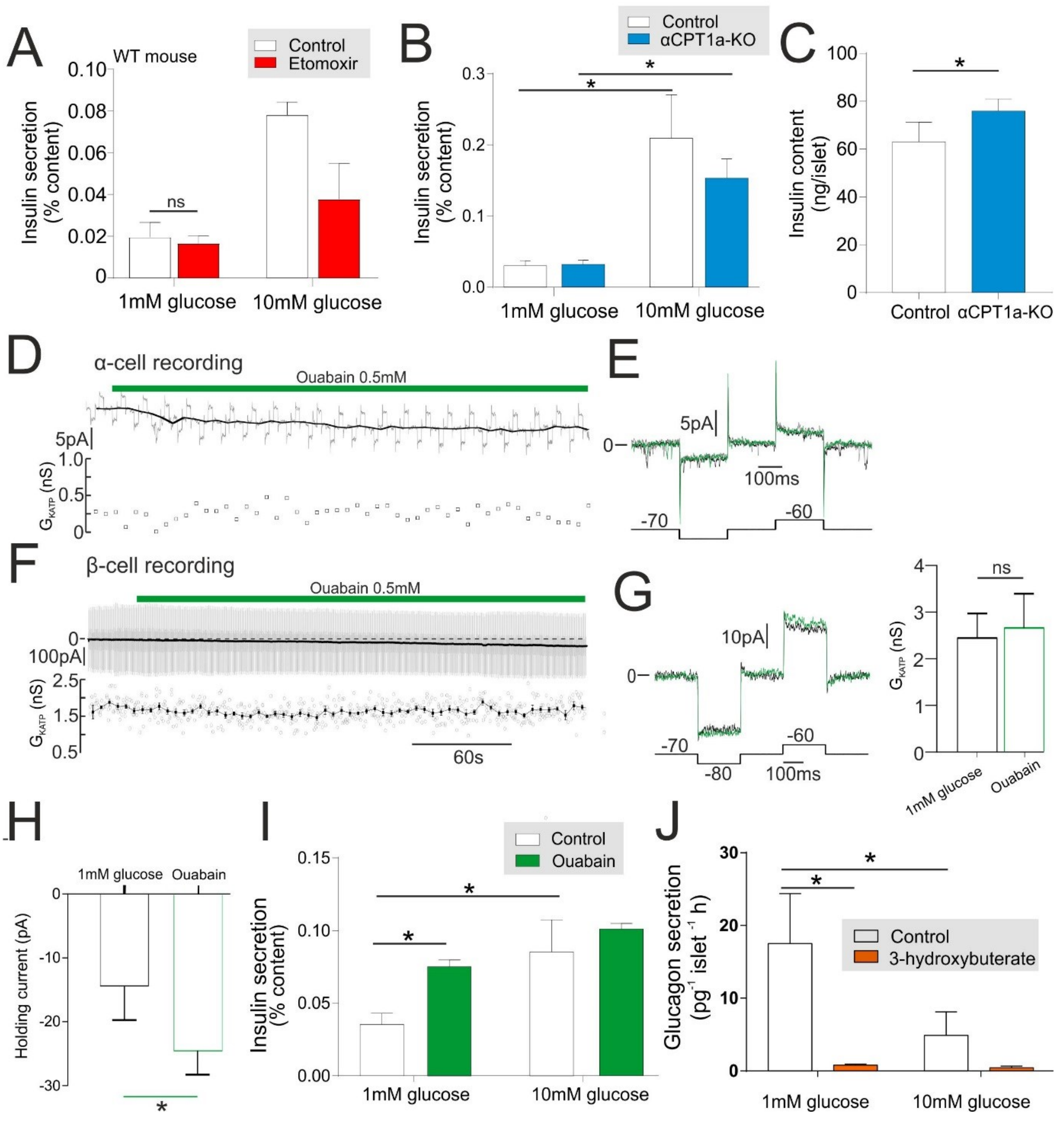

Supplementary Figure 1 (Related to Figure 1-3)

(A) Insulin secretion from WT islets at 1 and 10mM glucose with or without etomoxir (100 μM) reduced glucagon secretion (n=3).

(B) Insulin secretion from control and αCPT1a-KO mice at 1 and 10mM glucose (n=6).

(C) Insulin content from control and αCPT1a-KO islets (n=6).

(D) Continuously recording G_{KATP} in WT α-cells at 1mM glucose following ouabain (0.5mM) application.

(E) G_{KATP} in a WT α-cell at 1mM glucose with or without ouabain (0.5mM).

(F) Continuously recording G_{KATP} in a WT β-cell at 1mM glucose following ouabain (0.5mM) application

(G) G_{KATP} in a WT β-cell at 1mM glucose with or without ouabain

(I) Grouped G_{KATP} data from WT α-cell at 1mM glucose with or without ouabain (0.5mM) (3 cells from 3 mice).

(J) Glucagon secretion in WT mouse islets at 1 or 10mM glucose with or without 3-hydroxybuterate (0.5mM) (n=3).

All data are represented as mean ± SEM. Paired t-test with Tukey post-hoc or two-way ANOVA with Student Newman-Keuls post-hoc; * = $p<0.05$.

Figure S2

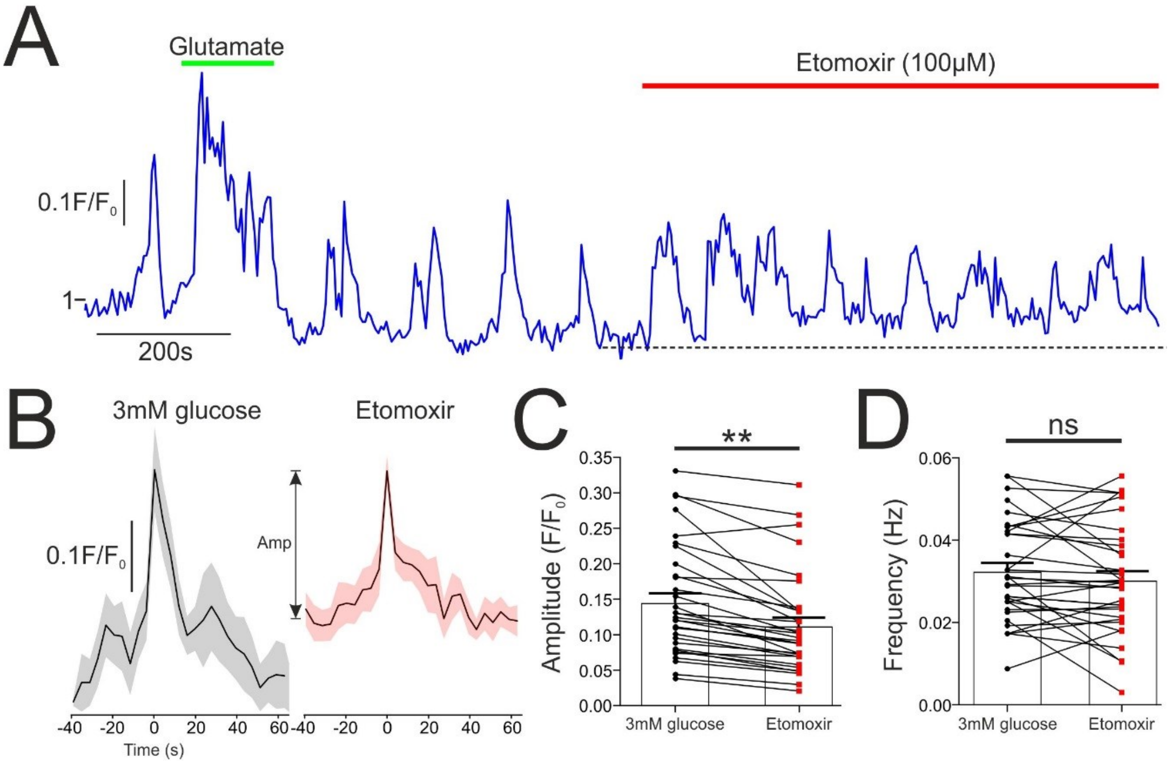

Supplementary Figure 2 (Related to Figure 4)

(A) Fluorescence intensity of intracellular calcium ($[Ca^{2+}]_i$) measured using Fluo-4 from intact pancreatic WT islets. Recordings were conducted in 3 mM glucose and 3 mM glucose with 100 µM etomoxir.

(B) For each experimental condition, the peak of each $[Ca^{2+}]_i$ spike was marked and a peak-triggered average waveform of $[Ca^{2+}]_i$ computed. The amplitude (Amp) of this average waveform was then measured.

(C) Amplitude of average $[Ca^{2+}]_i$ spike waveform (Amp) in 3 mM glucose with or without 100 µM etomoxir. (4 islets, 4 mice)

(D) Frequency of $[Ca^{2+}]_i$ spikes in 3 mM glucose with or without 100 µM etomoxir. (4 islets, 4 mice)

All data are represented as mean ± SEM. Paired t-test with Tukey post-hoc; ** = $p<0.01$.

Supplementary methods

Ethics
All animal experiments were conducted in accordance with the UK Animals Scientific Procedures Act (1986) and University of Oxford ethical guidelines, and were approved by the local Ethical Committees. Human pancreatic islets were isolated, with ethical approval and clinical consent, at the Diabetes Research and Wellness Foundation Human Islet Isolation Facility (OCDEM, Oxford, UK).

Animals
All animals were kept in a specific pathogen-free (SPF) facility under a 12:12 hour light:dark cycle at 22 °C, with unrestricted access to standard rodent chow and water. C57BL/6j mice used in this study are referred to as wild-type (WT) mice. To generate α-cell specific *Cpt1a* knockout mice (αCPT1a-KO), mice carrying a loxP insert flanking exons 11 and 12 of the *Cpt1a* gene (Schoors et al., 2015) were crossed with mice carrying a Cre recombinase under the control of the proglucagon promoter (Parker et al., 2012). Mice homozygous for the loxP allele were used as controls, and are referred to as such.

***In vivo* measurements of plasma glucose, glucagon and ketone body concentration**
Plasma glucose, glucagon and ketone body measurements were conducted *in vivo* on αCPT1a-KO and control mice in response to fasting. Experiments followed a strict time schedule to minimise experimental variation. Plasma glucose was measured with an Accu-Chek Aviva (Roche Diagnostic, UK) and plasma ketone bodies with a FreeStyle Libre system (Abbott Laboratories Ltd, Maidenhead, UK). Bovine aprotinin 2µl (4 TIU/ml) (Sigma-Aldrich, UK) was added to all plasma samples.
Fed plasma glucose: Mice were restrained and a tail vein sample of blood was used to measure fed plasma glucose and ketone bodies. At this stage, some mice were culled, and the liver was removed, snap frozen in liquid nitrogen and stored at -80 °C. Other mice were not culled, but instead used for subsequent *in vivo* studies.
Fasting studies: Mice were restrained and fed plasma glucose was measured as above. Mice were then individually caged for the 4 hour fasting period (8.30am-12.30pm) and given unrestricted access to water during this time. At the end of the fasting period, mice were restrained and a tail vein sample of plasma was used to measure plasma glucose and ketone bodies. Mice were then culled by cervical dislocation and trunk plasma collected in EDTA coated tubes. Blood sampels were kept on ice and imediately centrifuged at 2700 rpm for 10 min to obtain plasma. The plasma was then removed and stored at -80 °C.
Plasma glucagon measurements: Plasma samples (stored at -80 °C prior) were used to measure plasma glucagon. Measurements were conducted using a mouse glucagon assay system (Mercodia, Upsala, Sweden), according to the manufacturers protocol.

Pharmacological blockade of CPT1

Pharmacological blockade of CPT1 was achieved using the CPT1 inhibitor etomoxir (Sigma-Aldrich, UK). Concentrations of 25-200 µM have been used to block CPT1 activity in islet cells in other studies (Chen et al., 1994, Lehtihet et al., 2003). Prolonged exposure to high concentrations (1 mM) of etomoxir is known to increase ROS, deplete ATP and lead to cell death (Pike et al., 2011). Although our application of etomoxir was short (<1 hour), we opted for 100 µM to avoid this. This concentration of 100 µM was used throughout this study.

Isolation of pancreatic islets
Mice at 12-16 weeks of age were killed by cervical dislocation (Schedule 1 procedure). Pancreatic islets were isolated by liberase digestion followed by manual picking. Islets were used acutely and were, pending the experiments, maintained in tissue culture for <24 hour in RPMI 1640 (11879-020, Gibco, Thermo Fisher Scientific) containing 1% pennicillin/streptomycin (1214-122, Gibco, Thermo Fisher Scientific), 10%FBS (F7524-500G, Sigma-Aldrich) and 7.5mM glucose prior to the measurements.

Cell culture experiments
The αTC1-clone 6 cell line (αTC1-6; CRL-2934, ATCC), was cultured in RPMI 1640 (118279-020, Gibco, Thermo Fisher Scientific) containing 15mM Hepes (15630-56, Gibco, Thermo Fisher Scientific), 1% penicillin/streptomycin (15140-122, Gibco, Thermo Fisher Scientific), 10%FBS (10270-10, Gibco, Thermo Fisher Scientific) and 15mM glucose. For all experiments, cells were plated in 12 or 6 well plates, and, after 3 days of culture, glucose concentration was lowered to 5 mM over night and experiments were performed the next day. For knock down of *Cpt1a*, αTC1-6 cells were cultured for 2 days in standard medium and then

transfected with scrambled control siRNA (SR30004,Origene) or siRNA specifically targeting *Cpt1a* mRNA (J-042456-10-0002, Dharmacon Inc.). After 36 hour, medium was changed to culture medium containing 5 mM glucose and experiments were performed the next day. The efficiency of downregulation was assessed by Western blot.

Evaluation of protein expression
For evaluation of protein expression, αTC1-6 cells were lysed in Hepes KOH pH7.6 buffer containing 0.1% TritonX-100 (Sigma-Aldrich) and protease and phosphatase inhibitors (Roche, UK). Lysates were immedaitely sonicated and then stored at -80°C for further analysis. Protein content was determined by bicinchoninic acid assay (Thermo Fisher Scientific, UK).

Patch-clamp electrophysiology in islets
Mouse and human islets were used for patch-clamp electrophysiological recordings. These recordings (in intact islets) were performed at 33-34 ^{0}C using an EPC-10 patch-clamp amplifier and PatchMaster software (HEKA Electronics, Lambrecht/Pfalz, Germany). Currents were filtered at 2.9 kHz and digitized at > 10 kHz. A new islet was used for each recording. Membrane potential (V_M) recordings were conducted using the perforated patch-clamp technique, as previously described (De Marinis et al., 2010, Zhang et al., 2013). The pipette solution contained (in mM) 76 K_2SO_4, 10 NaCl, 10 KCl, 1 $MgCl_2{\cdot}6H_20$ and 5 Hepes (pH 7.35 with KOH). For these experiments, the bath solution contained (mM) 140 NaCl, 3.6 KCl, 10 Hepes, 0.5 $MgCl_2{\cdot}6H20$, 0.5 $Na_2H_2PO_4$, 5 $NaHCO_3$ and 1.5 $CaCl_2$ (pH 7.4 with NaOH). Amphotericin B (final concentration of 25mg/mL, Sigma-Aldrich) was added to the pipette solution to give electrical access to the cells (series resistance of <100 MΩ). α-cells were confirmed by their activity at 1 mM glucose and a logistic regression model that can identify cell type with 94% accuracy (Briant et al., 2017). Recordings were made during brief (5-10 min) exposure to etomoxir (100 μM), and action potential morphology was quantified and compared.

The morphology of action potentials and frequency of firing was calculated in MATLAB v. 6.1 (2000; The MathWorks, Natick, MA). In brief, a peak-find algorithm was used to detect action potentials. This was then used to calculate firing frequency in different experimental conditions. The peaks were then used as triggers to create a waveform-triggered average of membrane potential in different experimental conditions. The window chosen for this analysis was the entire experimental condition. This average action potential waveform was generated over a symmetric 50 ms window (25 ms before and after the trigger). The minimal membrane potential (V_{MIN}) was defined as the minimal membrane potential preceeding the action potential in this window. The action potential amplitude was defined as the amplitude of this average waveform. Analysis of action potential morphology in αCPT1a-KO and control mice was conducted 'blind' to mouse strain (blinding was conducted by an acknowledged independent researcher).

Quantitive imaging of ATP in islets
Time-lapse imaging of the ATP/ADP ratio in WT mouse islets was performed using 14× magnification on a Zeiss AxioZoom V16 microscope, as previously described (Adam et al., 2017). In brief, islets were transduced with an adenovirus delivering Perceval, a recombinant sensor of ATP/ADP. Groups of islets were imaged simultaneously 24 hour post-transduction at glucose concentrations as indicated, with single-cell resolution. Time-lapse images were collected every 30 s, and the bath solution was perifused at 60 μl/min at 34 °C. The bath solution was as described for patch-clamp electrophysiology.

Ca^{2+} imaging in islets

Time-lapse imaging of the intracellular Ca^{2+} concentration ($[Ca^{2+}]_i$) in WT mouse islets was performed on the inverted Zeiss AxioVert 200 microscope, equipped with the Zeiss LSM 510-META laser confocal scanning system, using a 40x/1.3 NA objective. Mouse islets were loaded with 6 mM of the Ca^{2+}-sensitive dye Fluo-4 for 90 min before being transfered to a recording chamber. Mice were then continuously perfused with bath solution (same solution as described for patch-clamp electrophysiology, above) at a rate of 200 μL/min. Fluo-4 was excited at 488 nm and fluorscence emission collected at 530 nm. The pinhole diameter was kept constant, and frames of 256x256 pixels were taken every 1-3 s. α-cells were identified by the presence of oscillations in $[Ca^{2+}]_i$ in low (3 mM) glucose and an excitatory response to glutamate.

Hormone secretion measurements
Islets: Islets isolated from WT, αCPT1a-KO and control mice were incubated for 1h in RPMI supplemented with 7.5 mM glucose in a cell culture incubator. Size-matched batches of 20 islets were pre-incubated in 0.2 ml

KRB with 2mg/ml BSA (S6003, Sigma-Aldrich) and 3 mM glucose for 1 hour in a water-bath at 37 ^{0}C. Following this islets were sequentially subjected to 0.2 ml KRB with 2 mg/ml BSA with 1 mM or 10 mM glucose for 1 hour. For Ketone body experiments islets were subjected to with 1 mM or 10 mM glucose with or without 0.5mM 3-hydroxybuterate for 1 hour After each incubation, the supernatant was removed, quickly frozen and stored at -80 ^{0}C. At the end of the experiment the islets were lysed in 0.1 ml of HCl:ethanol (1:15) and samples were sonicated and stored at -80 ^{0}C.
αTC1-6 cells: on the day of the experiment, cells were pre-incubated for 1 hour at 0 mM glucose KRB, with or without etomoxir (100 µM). The cells were then incubated for 1 hour in KRB containing either 1 or 10 mM glucose. After 1 hour, the supernatant was removed and centrifuged at 800 g, 4 °C for 10 min, to remove dead cell debris. The supernatant was transferred to clean eppendorf tubes and stored at -80 °C. Cells in the plate were lysed with 0.5 ml of HCl:ethanol (1:15), sonicated and stored at -80°C. Glucagon and insulin from secretion and content samples were measured using MSD mouse insulin/glucagon duplex sandwich ELISA (Mesoscale Discovery, USA), according to the manufacturers protocol.

Pancreas perfusion: Measurements of glucagon secretion was performed using *in situ* pancreatic perfusion. Briefly, the aorta was ligated above the coeliac artery and below the superior mesenteric artery and then cannulated. The pancreas was perfused with KRB containing varying concentrations of glucose in the following order 4, 6 and then 10 mM glucose at a speed of 0.240 ml/min using an Ismatec Reglo Digital MS2/12 peristaltic pump. The perfusate was maintained at 37°C using a Warner Instruments temperature control unit TC-32 4B in conjunction with a tube heater (Warner Instruments P/N 64-0102) and a Harvard Apparatus heated rodent operating table. The effluent was collected in intervals of 2 min in to 96 well plate on ice containing aprotenin. Samples were subsequently stored at -80°C. Glucagon content in perfusate were measured using mouse glucagon RIA (Euro-diagnostica, Sweden), according to the manufacturers protocol.

FFA oxidation measurements
αTC1-6 cells were cultured over night in RPMI culture medium containing 5 mM glucose. On the day of the experiment, the cells were incubated in KRB with 0 mM glucose and the relevant treatment (e.g. etomoxir). The cells were then exposed to 0.3 mM palmitate containing 0.22Mbeq H^3palmitate for 1 hour. The supernatant was then subjected to a Falkes extraction and the aquaeus phase assayed for H^3 content. From this, β-oxidation was calculated and normalised to cell count.

Immunohistochemistry and immunoblotting
Mouse or human pancreata or islets were fixed in 0.4% PFA 10% neutral-buffered formalin, dehydrated and processed for paraffin wax embedding and sectioning (3µm).

Immunostaining was performed using antibodies to CPT1a (ab128568, ABCAM, Cambridge, UK), CPT1b (ABCAM, Cambridge, UK), glucagon (ABCAM, Cambridge, UK) or glucagon (Sigma-Aldrich). CPT1b and glucagon staining was visualised using anti rabbit Alexafluor 488 (Molecular Probes, Thermo Fisher Scientific) or anti rabbit FITC secondary antibody. CPT1a staining was enhaced using VectaFluor™ Excel Amplified DyLight® 594 Anti-Mouse IgG Kit (DK-2594, Vectorlabs). Images were acquired with a Zeiss LSM510 META confocal imaging system.

For immunoblotting, cell lysates were diluted to a concentration of 1µg/ml in 2x Sample Buffer (BioRad) with β-mercaptoethanol (Sigma-Aldrich). After addition of the Laemmli sample buffer the proteins were separated usign SDS-PAGE, transferred to a PVDF membranes and visualised using antibodies to CPT1a, G6PC, β-actin (AbCam, UK), PEPCK (Cayman Chemical), calnexin (Millipore Merk) and HRP-conjugated secondary antibodies (Thermo Fisher Scientific, Rugby, UK). Blots were developed using ECL detection reagent (BioRad), images acquired on a ChemiDoc imager (Bio-Rad) and analysed using ImageLab software (BioRad). All data were normalised to either calnexin or β-actin and expressed as fold change of control.

Mathematical model of α-cell membrane potential
All simulations were conducted in the simulation environment NEURON using CVODE and a 25 µs timestep (Carnevale and Hines, 2006). The equation describing membrane potential (V_M) in the α-cell model is:

$$C_{cell}\frac{dV_M}{dt} = -(I_{CaL} + I_{CaN} + I_{CaT} + I_{Na} + I_K + I_{KATP} + I_{KA} + I_L) \qquad (1)$$

where: C_{cell} is the cell capacitance; I_{CaL}, I_{CaN} and I_{CaT} are L-, N-, and T-type voltage-dependent Ca^{2+} currents, respectively; I_{Na} is a voltage-dependent Na^+ current; I_K is a delayed rectifier K^+ current; I_{KA} is an A-type

voltage-dependent K$^+$ current; $I_{K(ATP)}$ is an ATP-sensitive K$^+$ current; I_L is a leak current. This model is described in detail in (Briant et al., 2018). In addition, we modelled the current due to the Na$^+$-K$^+$ pump (Na$^+$/K$^+$-ATPase), I_{pump}. The equations describing this current are

$$I_{pump} = \frac{\overline{I_{pump}}}{1 + \left({k_m}/{[Na^+]_i}\right)^n}$$

as described in Canavier (1999). This current writes to the transmembrane Na$^+$ and K$^+$ currents in the NEURON environent, as follows:

$$I_{Na} = 3 \cdot I_{pump}$$
$$I_K = -2 \cdot I_{pump}$$

To mimic reduced ATP supply to the Na$^+$-K$^+$ pump, we reduced the maximal pump current, $\overline{I_{pump}}$.

Glucagon release was modelled by using the 4 variable system described by Yamada and Zucker (1992) and later simplified by Destexhe et al. (1994). In brief, the intracellular calcium concentration $[Ca^{2+}]$ was modelled as the sum of calcium fluxes due to the total calcium current ($I_{Ca} = I_{CaL} + I_{CaN} + I_{CaT}$) and a calcium buffering term:

$$\frac{d[Ca^{2+}]}{dt} = \frac{2I_{Ca}}{F_d \cdot d} + \frac{([Ca^{2+}]_0 - [Ca^{2+}])}{\tau} \quad (2)$$

Here, calcium is buffered to $[Ca^{2+}]_0$ with time-constant τ, F_d is Faradays constant and d is the depth of the calcium domain. This calcium concentration drives a system of differential equations describing glucagon vesicle dynamics:

$$\frac{d[F_A]}{dt} = k_b([F_{max}] - [F_A] - [V_A])[Ca^{2+}]^4 - k_u[F_A] - k_1[F_A][V] + k_2[V_A] \quad (3)$$

$$\frac{d[V_A]}{dt} = k_1[F_A][V] - (k_2 + k_3)[V_A] \quad (4)$$

$$\frac{d[GCG]}{dt} = Nk_3[V_A] - k_h[GCG] \quad (5)$$

Here, calcium ions are assumed to reversibly bind to a fusion protein F. Four calcium ions bind to this protein at a rate k_b, activating it. The concentration of activiated fusion protein is $[F_A]$, coming from a pool of inactivated proteins with concentration $[F_{max}]$. The reverse process has an unbinding rate k_u. An activated fusion protein binds to a vesicle (V) at a rate k_1, activated it (V_A). This process is reversible with unbind rate k_2. The concentrations of inactivated and activated vesicles are $[V]$ and $[V_A]$, respectively. Destexhe et al. (1994) simplified this system by assuming that there exists an inexhaustible pool of inactivated vesicles, ready for activation. In particular, $[V]$ is constant and not depleted. This assumption is adopted. An activated vesicle is then able to fuse to the membrane of the cell, and release its contents. An activated vesicle releases N molecules of glucagon (GCG) at a rate k_3. The concentration of glucagon released is $[GCG]$. This is depleted in the extracellular space by diffusion, degradation and reuptake at a rate k_h.

Statistical tests

All data are reported as mean ± S.E.M., unless otherwise stated. Statistical significance was defined as $p<0.05$. All statistical tests were conducted in Prism5 (GraphPad Software, San Diego, CA). For two groupings, a *t*-test was conducted. If the data were non-parametric, a Mann-Whitney test was conducted. For more than two groupings, a one-way ANOVA was conducted. If there were two independent variables, a two-way ANOVA was conducted. If the data passed normality criteria (D'Agostino's test of normality and Bartlett's test of equal variances), a parametric test was conducted with the appropriate post hoc test (Tukey or Student Neumann Keuls). If the normality criteria were not met, a Kruskal–Wallis test with Dunn's multiple comparison test was conducted.

References

ADAM, J., RAMRACHEYA, R., CHIBALINA, M. V., TERNETTE, N., HAMILTON, A., TARASOV, A. I., ZHANG, Q., REBELATO, E., RORSMAN, N. J. G., MARTIN-DEL-RIO, R., LEWIS, A., OZKAN, G., DO, H. W., SPEGEL, P., SAITOH, K.,

KATO, K., IGARASHI, K., KESSLER, B. M., PUGH, C. W., TAMARIT-RODRIGUEZ, J., MULDER, H., CLARK, A., FRIZZELL, N., SOGA, T., ASHCROFT, F. M., SILVER, A., POLLARD, P. J. & RORSMAN, P. 2017. Fumarate Hydratase Deletion in Pancreatic beta Cells Leads to Progressive Diabetes. *Cell Rep,* 20**,** 3135-3148.

BRIANT, L. J., ZHANG, Q., VERGARI, E., KELLARD, J. A., RODRIGUEZ, B., ASHCROFT, F. M. & RORSMAN, P. 2017. Functional identification of islet cell types by electrophysiological fingerprinting. *J R Soc Interface,* 14.

BRIANT, L. J. B., REINBOTHE, T. M., SPILIOTIS, I., MIRANDA, C., RODRIGUEZ, B. & RORSMAN, P. 2018. delta-cells and beta-cells are electrically coupled and regulate alpha-cell activity via somatostatin. *J Physiol,* 596**,** 197-215.

CANAVIER, C. C. 1999. Sodium dynamics underlying burst firing and putative mechanisms for the regulation of the firing pattern in midbrain dopamine neurons: a computational approach. *J Comput Neurosci,* 6**,** 49-69.

CARNEVALE, N. T. & HINES, M. L. 2006. *The NEURON book,* Cambridge, UK ; New York, Cambridge University Press.

CHEN, S., OGAWA, A., OHNEDA, M., UNGER, R. H., FOSTER, D. W. & MCGARRY, J. D. 1994. More direct evidence for a malonyl-CoA-carnitine palmitoyltransferase I interaction as a key event in pancreatic beta-cell signaling. *Diabetes,* 43**,** 878-83.

DE MARINIS, Y. Z., SALEHI, A., WARD, C. E., ZHANG, Q., ABDULKADER, F., BENGTSSON, M., BRAHA, O., BRAUN, M., RAMRACHEYA, R., AMISTEN, S., HABIB, A. M., MORITOH, Y., ZHANG, E., REIMANN, F., ROSENGREN, A. H., SHIBASAKI, T., GRIBBLE, F., RENSTROM, E., SEINO, S., ELIASSON, L. & RORSMAN, P. 2010. GLP-1 inhibits and adrenaline stimulates glucagon release by differential modulation of N- and L-type Ca2+ channel-dependent exocytosis. *Cell Metab,* 11**,** 543-53.

DESTEXHE, A., MAINEN, Z. F. & SEJNOWSKI, T. J. 1994. Synthesis of models for excitable membranes, synaptic transmission and neuromodulation using a common kinetic formalism. *J Comput Neurosci,* 1**,** 195-230.

LEHTIHET, M., WELSH, N., BERGGREN, P. O., COOK, G. A. & SJOHOLM, A. 2003. Glibenclamide inhibits islet carnitine palmitoyltransferase 1 activity, leading to PKC-dependent insulin exocytosis. *Am J Physiol Endocrinol Metab,* 285**,** E438-46.

PARKER, H. E., ADRIAENSSENS, A., ROGERS, G., RICHARDS, P., KOEPSELL, H., REIMANN, F. & GRIBBLE, F. M. 2012. Predominant role of active versus facilitative glucose transport for glucagon-like peptide-1 secretion. *Diabetologia,* 55**,** 2445-2455.

PIKE, L. S., SMIFT, A. L., CROTEAU, N. J., FERRICK, D. A. & WU, M. 2011. Inhibition of fatty acid oxidation by etomoxir impairs NADPH production and increases reactive oxygen species resulting in ATP depletion and cell death in human glioblastoma cells. *Biochim Biophys Acta,* 1807**,** 726-34.

SCHOORS, S., BRUNING, U., MISSIAEN, R., QUEIROZ, K. C. S., BORGERS, G., ELIA, I., ZECCHIN, A., CANTELMO, A. R., CHRISTEN, S., GOVEIA, J., HEGGERMONT, W., GODDE, L., VINCKIER, S., VAN VELDHOVEN, P. P., EELEN, G., SCHOONJANS, L., GERHARDT, H., DEWERCHIN, M., BAES, M., DE BOCK, K., GHESQUIERE, B., LUNT, S. Y., FENDT, S.-M. & CARMELIET, P. 2015. Fatty acid carbon is essential for dNTP synthesis in endothelial cells. *Nature,* 520**,** 192-197.

YAMADA, W. M. & ZUCKER, R. S. 1992. Time course of transmitter release calculated from simulations of a calcium diffusion model. *Biophys J,* 61**,** 671-82.

ZHANG, Q., RAMRACHEYA, R., LAHMANN, C., TARASOV, A., BENGTSSON, M., BRAHA, O., BRAUN, M., BRERETON, M., COLLINS, S., GALVANOVSKIS, J., GONZALEZ, A., GROSCHNER, L. N., RORSMAN, N. J., SALEHI, A., TRAVERS, M. E., WALKER, J. N., GLOYN, A. L., GRIBBLE, F., JOHNSON, P. R., REIMANN, F., ASHCROFT, F. M. & RORSMAN, P. 2013. Role of KATP channels in glucose-regulated glucagon secretion and impaired counterregulation in type 2 diabetes. *Cell Metab,* 18**,** 871-82.
